# Advances in Atroposelectively De Novo Synthesis of Axially Chiral Heterobiaryl Scaffolds

**DOI:** 10.3390/molecules27238517

**Published:** 2022-12-03

**Authors:** Xiaoke Zhang, Ya-Zhou Liu, Huawu Shao, Xiaofeng Ma

**Affiliations:** 1 Natural Products Research Centre, Chengdu Institute of Biology, Chinese Academy of Sciences, Chengdu 610041, China; 2 Central Laboratory, Chongqing University Fu Ling Hospital, Chongqing 408000, China

**Keywords:** de novo synthesis, heterobiaryl, axially chiral, asymmetric synthesis

## Abstract

Axially chiral heterobiaryl frameworks are privileged structures in many natural products, pharmaceutically active molecules, and chiral ligands. Therefore, a variety of approaches for constructing these skeletons have been developed. Among them, de novo synthesis, due to its highly convergent and superior atom economy, serves as a promising strategy to access these challenging scaffolds including C-N, C-C, and N-N chiral axes. So far, several elegant reviews on the synthesis of axially chiral heterobiaryl skeletons have been disclosed, however, atroposelective construction of the heterobiaryl subunits by de novo synthesis was rarely covered. Herein, we summarized the recent advances in the catalytic asymmetric synthesis of the axially chiral heterobiaryl scaffold via de novo synthetic strategies. The related mechanism, scope, and applications were also included.

## 1. Introduction

**Figure 1:**
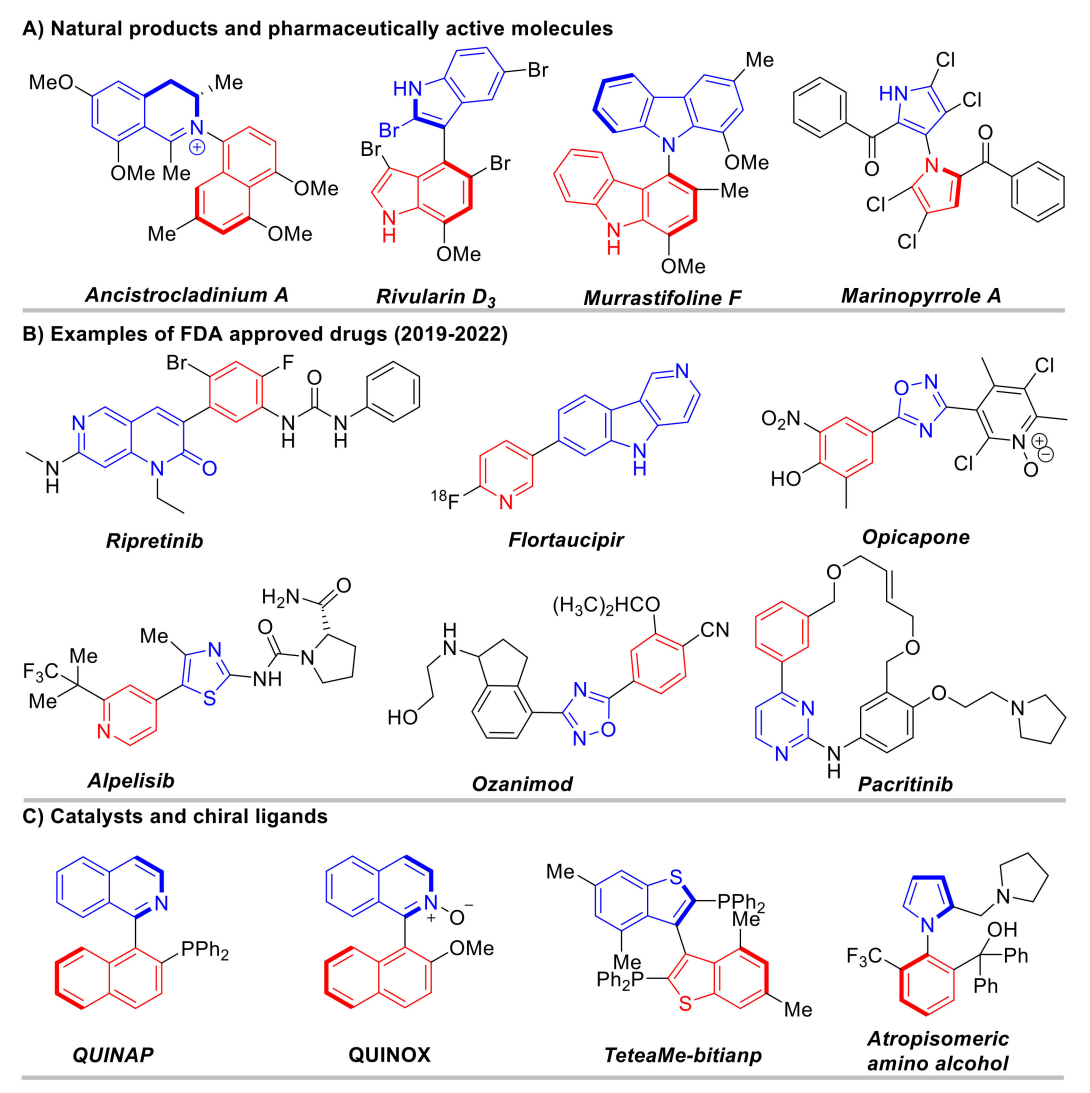
Representative heterobiaryl scaffolds in natural products, drug molecules, catalysts, and chiral ligands.

A common form of axial chirality is atropisomerism, which results from the inhibition of σ bond rotation caused by steric hindrance or the electronic effect of the flanking substituents [1,2]. When the half-life of interconversion is at least 1000 s under the specific temperature, and the rotational barrier is higher than 23.3 kcal mol^−1^, the two atropisomers could be separated [3]. Although Christie and Kenner resolved the stable isomers of 6,6′-dinitro-2,2′-diphenic acid as early as 1922 [4], the importance of atropisomerism was only realized after the pioneering work of Noyori and Takaya in 1980, in which atropisomeric chiral BINAP (2,2′-bis(diphenylphosphino)-1,1′-binaphthyl) was successfully applied as a ligand for rhodium-catalyzed asymmetric hydrogenation of α-(acylamino) acrylic acids [4,5]. Since then, atropisomerically chiral catalysis has received remarkable attention, and axially chiral heterobiaryl frameworks have, thus, been recognized as a privileged structure in many natural products, pharmaceutically active molecules, and chiral ligands (Figure 1) [6,7,8,9,10,11,12,13]. For example, natural products with the heterobiaryl skeleton, such as *Ancistrocladinium A*, *Rivularin D_3_*, *Murrastifoline F*, and *Marinopyrrole A*, were isolated from marine blue-green alga *Rivularia firma*, *Murraya koenigii*, and actinomycete strain CNQ-418, and some of these products exhibit excellent activities in antimicrobial bioassays (Figure 1A) [14,15,16]. In addition, axial heterobiaryl compounds are also present in many FDA-approved drugs, and they have been proven to have a positive contribution to pharmacokinetics including absorption, distribution, metabolism, excretion, and potency via interaction with the target protein, which made heterobiaryl molecules more and more prevalent in drug discovery and drug development (Figure 1B) [17,18,19,20,21]. In addition, axially chiral heterobiaryl subunits, such as isoquinoline containing phosphine and atropisomeric isoquinoline *N*-oxide, have been successfully employed as powerful chiral ligands to construct various chiral molecules through transition-metal catalyzed asymmetric transformation (Figure 1C) [22,23,24,25,26,27].

**Figure 2:**
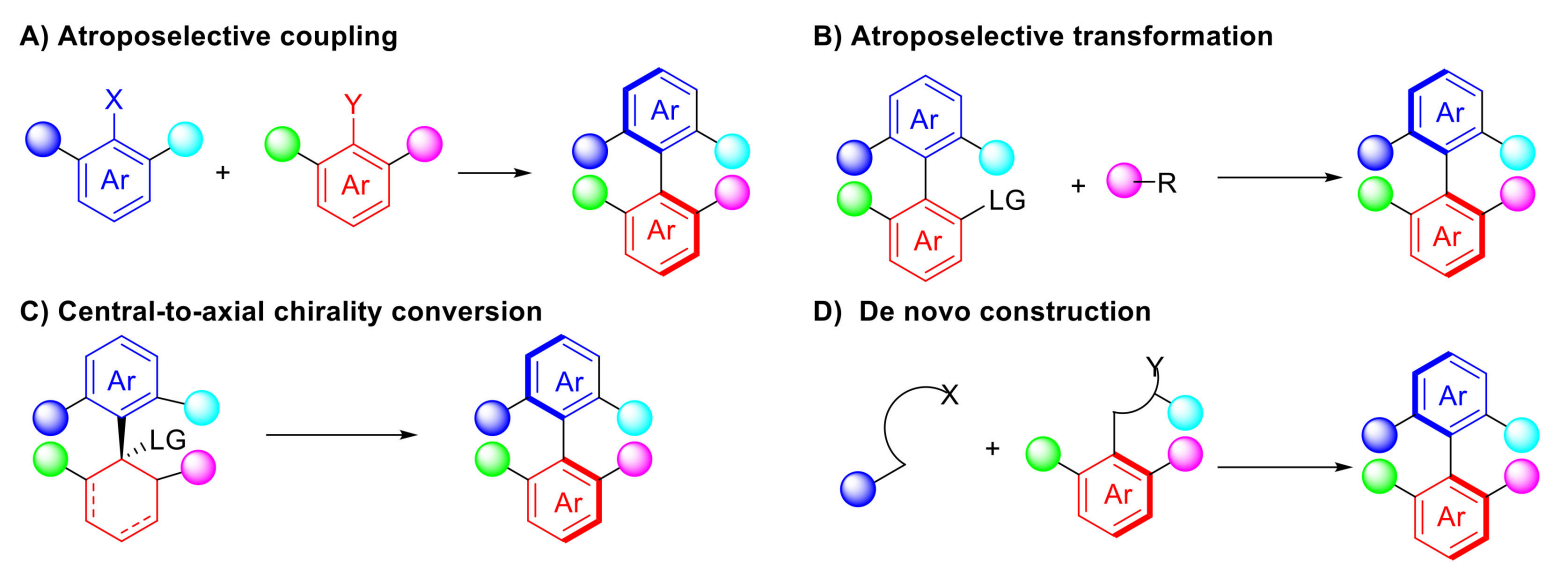
Representative synthesis method toward the heterobiaryl scaffold. Ar = aromatic ring or heteroaromatic ring; LG = leaving group.

In view of these significant applications, efficient approaches for synthesizing these axially chiral heterobiaryl skeletons are highly desirable [12,28,29,30]. However, it is well understood that construction of the axially chiral heterobiaryl scaffolds is very challenging, due to the following issues: (i) when heteroaryl sub-units are used as the coupling partners in the cross-coupling or oxidative dimerization reactions, the strong coordination ability of heteroatoms has a non-negligible impact on these transformations [30,31]; (ii) the low steric hindrance of nitrogen lone pair can lead to the poor conformational stability of some pyridine-derived atropisomers [12]; (iii) in addition to configurational instability and the low rotation barrier, the increased distance between the ortho-substituents around the axis makes the atroposelective synthesis of five-membered heterobiaryl molecules more challenging [32]. Nevertheless, a series of outstanding strategies to prepare axially chiral biaryls have been developed. These diverse methodologies can be divided into four main categories (Figure 2): (A) atroposelective coupling of two aromatic sub-units [33,34,35,36], (B) transformation of prochiral heterobiaryl molecules into axially chiral skeletons [37,38,39,40], (C) central-to-axial chirality conversion [41,42,43], and (D) de novo construction of axially chiral molecules [44,45,46]. To date, several elegant reviews have been disclosed, in which the authors focused on the atroposelective coupling of two units [47,48], atroposelective transformation of prochiral heterobiaryls [11,49,50,51], and central-to-axial chirality conversion [52,53]. Although de novo synthesis of axially chiral heterobiaryl exhibits highly convergent and superior atom economy, atroposelective construction of the heterobiaryl subunits by de novo synthesis was rarely covered. Moreover, the broader substitution patterns of de novo synthesis could increase the structural diversity, which is in turn, conducive to the application of heterobiaryl molecules in the development of chiral ligands for asymmetric synthesis and bioactive molecules for drug discovery. Thus, the synthesis of axially chiral heterobiaryl compounds from acyclic precursors through cyclization reaction has gained more and more attention recently.

In this review, we summarized the recent research progress and trend of axial chiral heteroaryl synthesis by means of de novo construction. The construction of heterobiaryl via two or more steps [54,55,56,57] and the synthesis of nonbiaryl atropisomers containing a heteroaryl scaffold [58,59,60] are out of the scope of this review. This mini-review is divided into the following parts: (1) transition metals catalyzed de novo synthesis of axially chiral heterobiaryl scaffolds; (2) organic small molecules catalyzed de novo synthesis of axially chiral heterobiaryl scaffolds.

## 2. The De Novo Synthesis of Axially Chiral Heterobiaryl Scaffolds Catalyzed by Transition Metal Catalysts

This section covers: (1) Intermolecular cyclization and cycloaddition reactions catalyzed by transition metal catalysts; (2) Transition metals-catalyzed intramolecular cyclization reactions.

### 2.1. Intermolecular Cyclization and Cycloaddition Reactions Catalyzed by Transition Metal Catalysts

#### 2.1.1. Enantioselective Synthesis of Five-Membered Ring Axially Chiral Heteroaromatic Skeletons

**Scheme 1:**
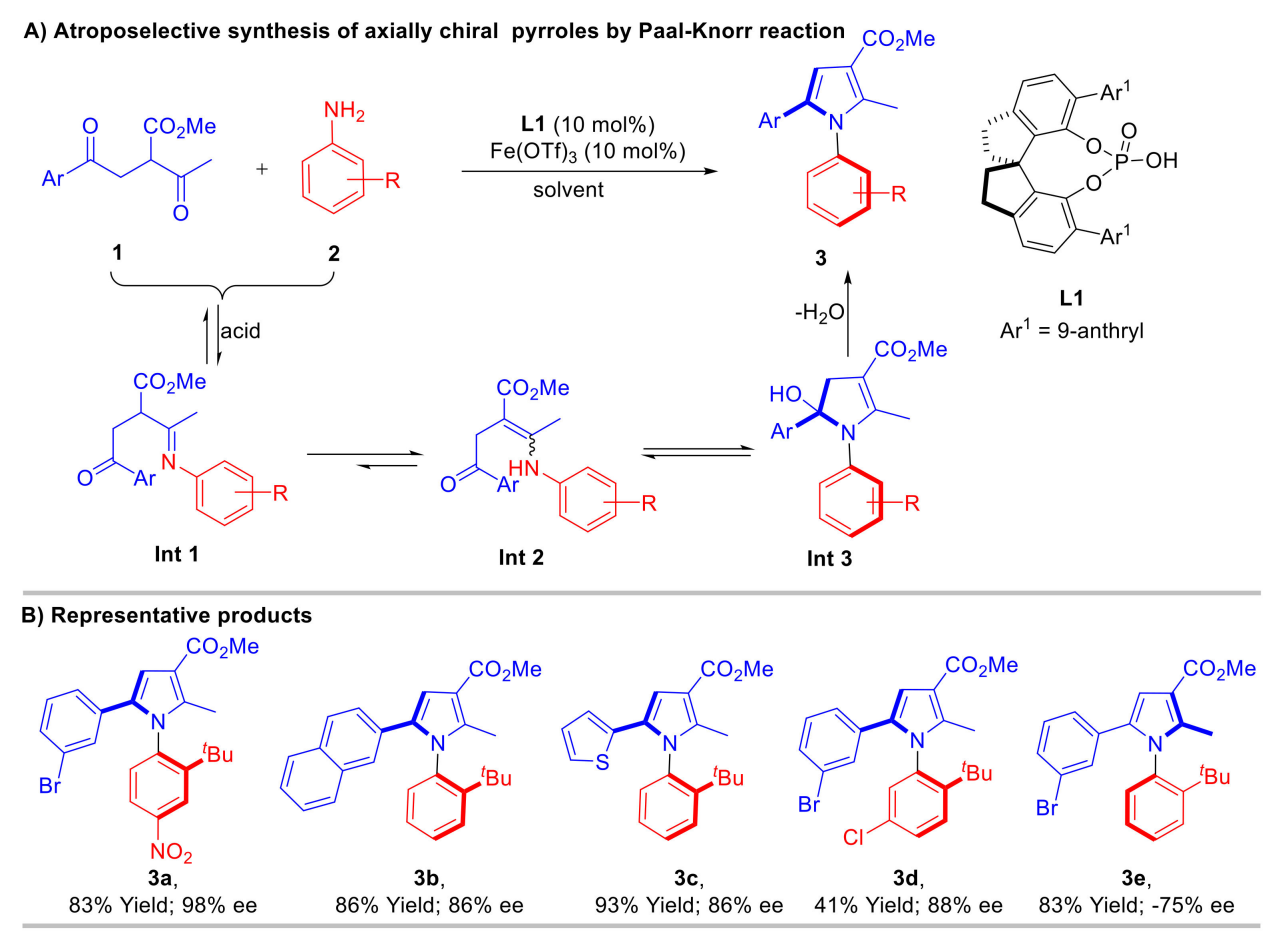
The synthesis of arylpyrroles by a catalytic asymmetric Paal–Knorr reaction.

The conformational instability and lower rotation barrier make the synthesis of atropisomeric species bearing the five-membered rings challenging, nevertheless, some elegant methodologies have been reported in recent years. In 2017, Tan’s group discovered an efficient strategy for highly atroposelective synthesis of enantiomerically pure aryl pyrroles through an asymmetric Paal–Knorr reaction between 1,4-dione **1** and substituted anilines **2** catalyzed by the combination of chiral phosphoric acid and Fe(OTf)_3_ (Scheme 1) [61]. Although using chiral phosphoric acid as a single catalyst only resulted in moderate enantioselectivity, the ee value was improved from 78% to 88% when Fe(OTf)_3_ was added as a Lewis acidic catalyst to activate phosphoric acid [62]. Significantly, an unexpected solvent-dependent inversion of the enantioselectivity was also discovered in this conversion. When the solvent was switched from CCl_4_ to MeOH, the enantioselectivity was changed from 72% ee to −52% ee under otherwise identical conditions. In addition, various functionalized aryl pyrroles could be accessed through enamine intermediate **Int 2**, followed by acid-catalyzed dehydrative cyclization (**3a**–**3d**). The presence of **Int 2** formed in the reaction mixture was confirmed by NMR and HRMS. Furthermore, axially chiral phosphine ligand **3d** could also be achieved in 88% ee through simply changing tertiary butyl (t-Bu) groups in amine **2** into diphenylphosphine analogs.

**Scheme 2:**
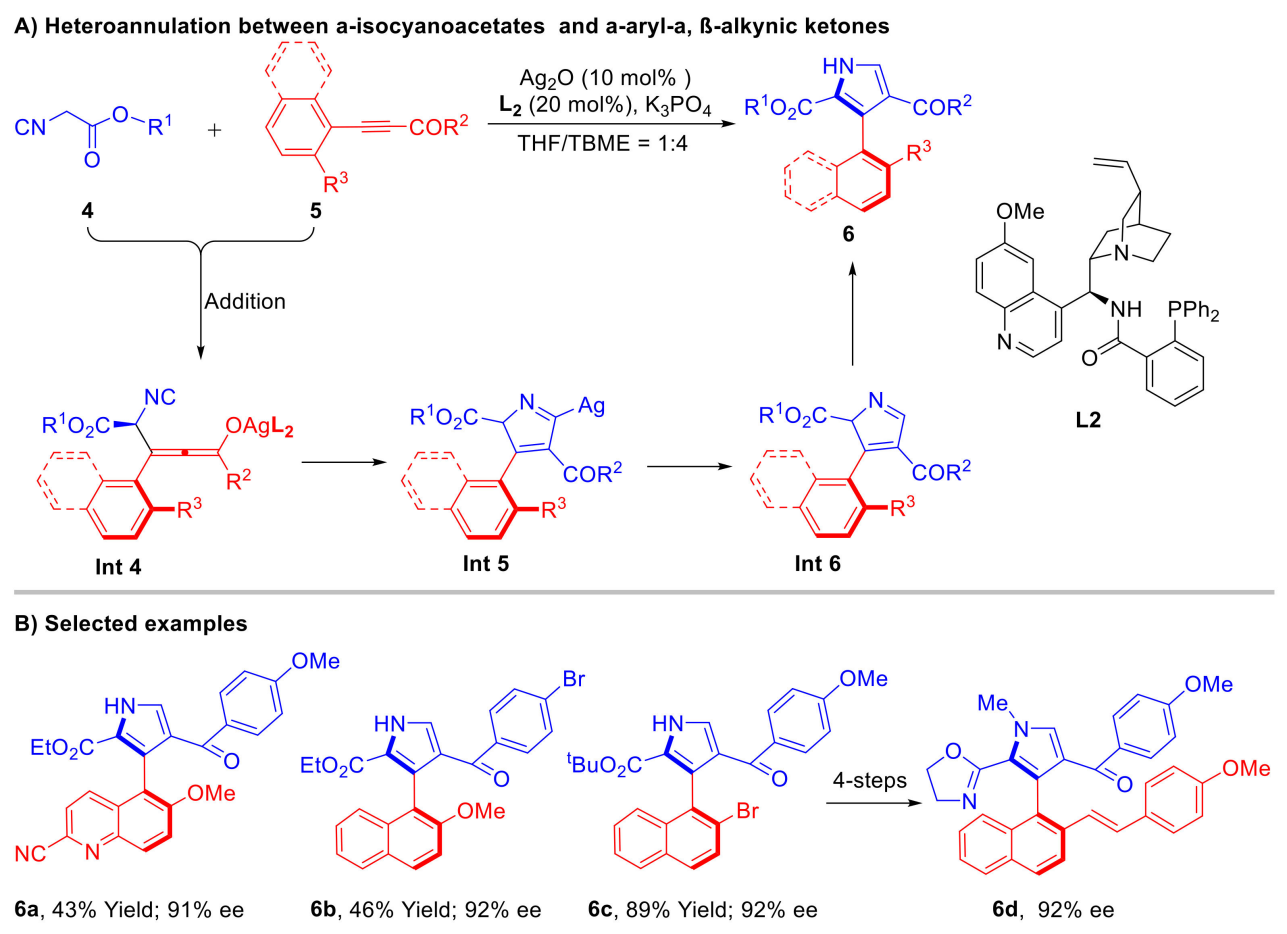
The synthesis of enantioenriched axially chiral 3-arylpyrroles.

Inspired by Tan’s work, Zhu and co-workers revealed a catalytic enantioselective heteroannulation between α-isocyanoacetates **4** and α-aryl-α,β-alkynic ketones **5** catalyzed by Ag_2_O in the presence of a cinchona-based phosphine ligand **L2**, which afforded axially chiral 3-arylpyrroles **6** in high yields with excellent enantioselectivities (Scheme 2) [63]. It is worth noting that **6a** bearing a quinoline system could be furnished in 91% ee. The authors proposed a possible mechanism in which nucleophilic addition of **4** and **5** provided the allene **Int 4**, which was followed by intramolecular cyclization to generate **Int 5**. Subsequently, the protonation of **Int 5** afforded **Int 6**. Finally, the 1,5-H shift of **Int 6** offered the corresponding axially chiral 3-arylpyrroles **6**. To demonstrate the utility of the reaction, the chiral olefin oxazole ligand **6d**, which could be potentially used for a catalytic asymmetric conjugate addition reaction, was prepared from the aryl pyrrole **6c** through a four-step transformation.

**Scheme 3:**
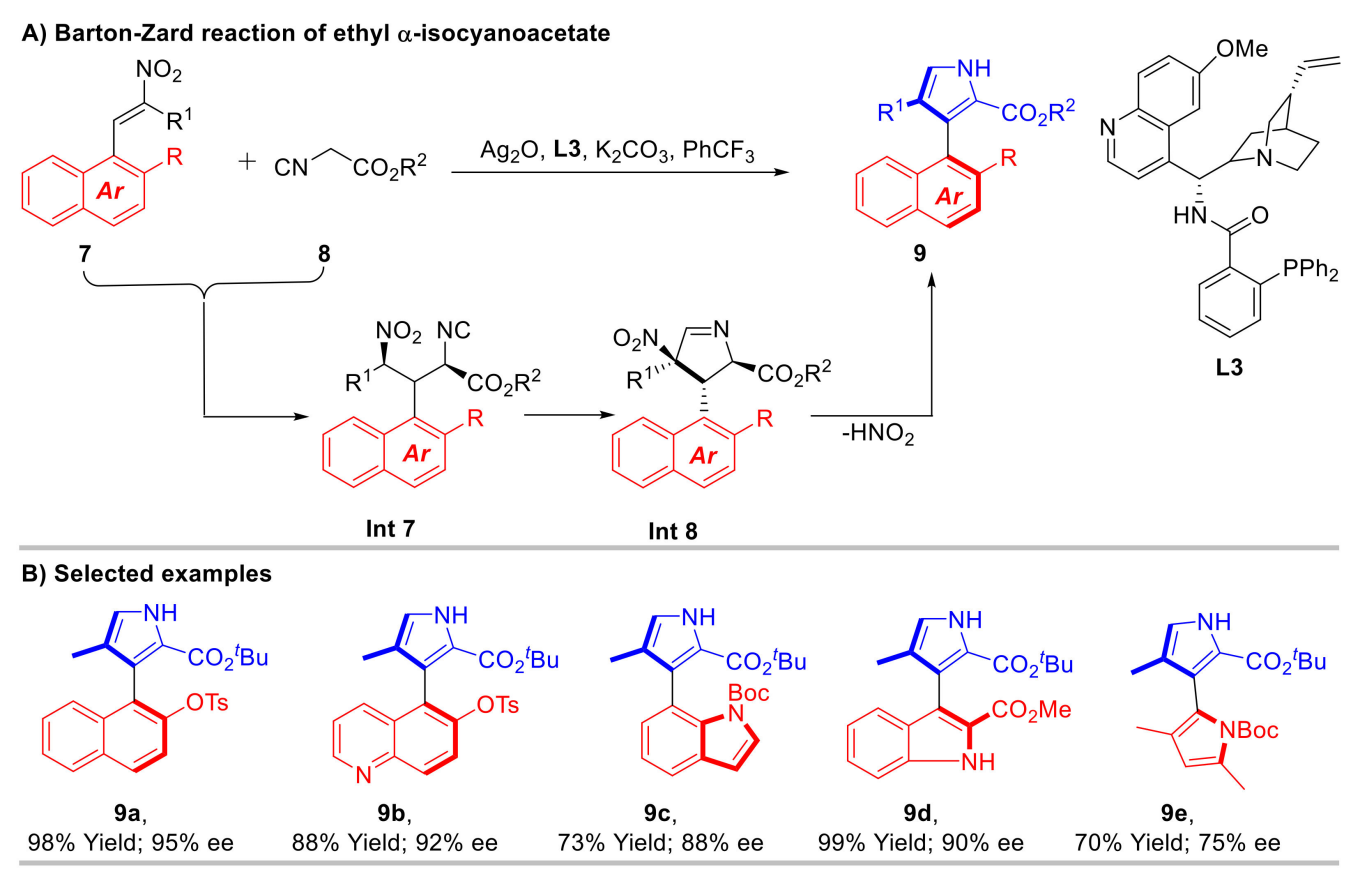
Access to 3-pyrrole-containing axially chiral skeletons catalyzed by Ag_2_O and **L3**.

Chen and co-workers reported the synthesis of 3-pyrrole-containing axially chiral scaffolds via an atropenantioselective Barton−Zard reaction of ethyl α-isocyanoacetate **8** and nitroolefin **7** catalyzed by Ag_2_O in the presence of cinchona-derived phosphine ligand **L3** (Scheme 3) [64]. This work presented a central-to-axial chirality transfer process, in which Michael addition of **8** to β-nitrostyrenes **7** generated **Int 7** with three central stereogenic centers. Subsequently, intramolecular cyclization and aromatization via the elimination of HNO_2_ could afford the axially chiral compound **9**.

**Scheme 4:**
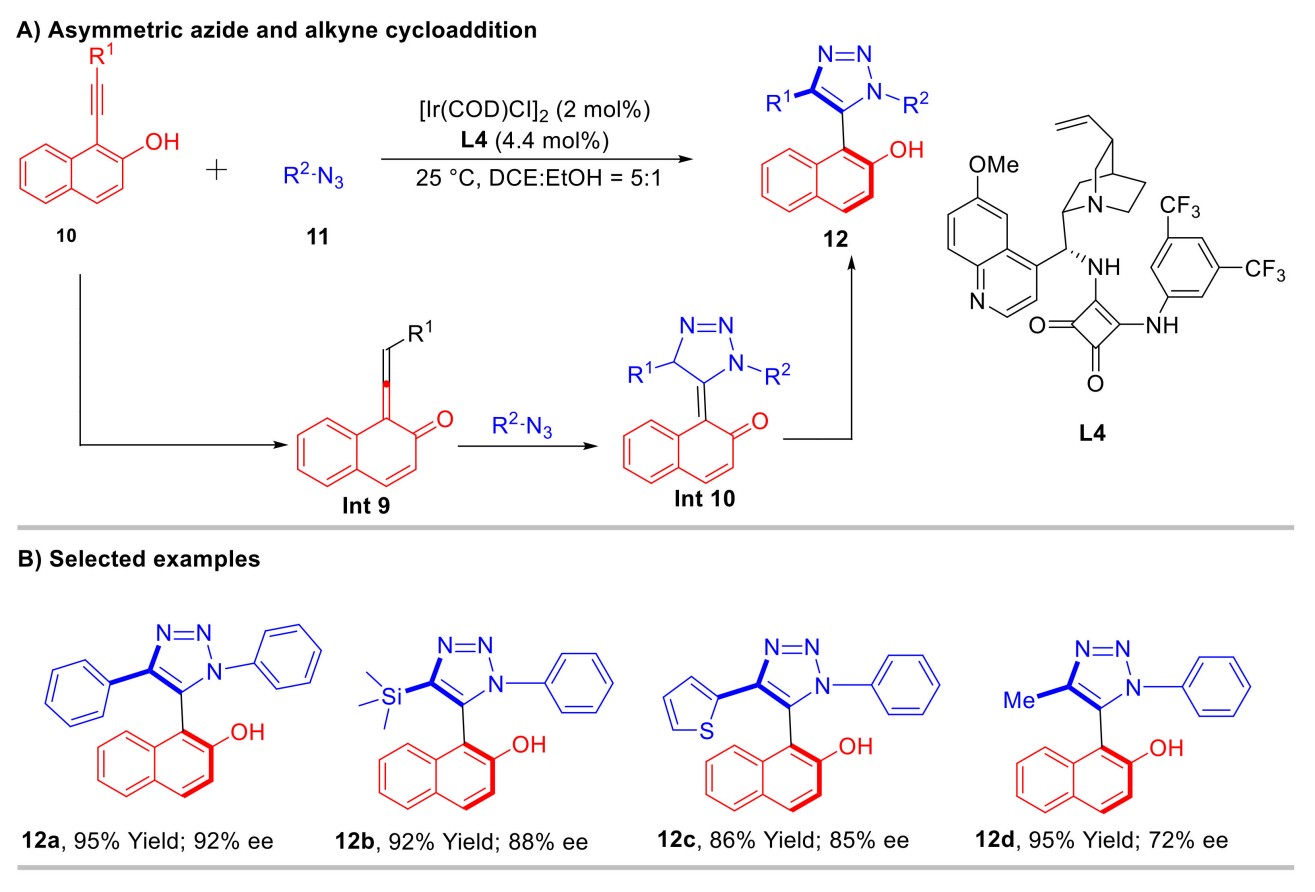
Asymmetric azide–alkyne cycloaddition with Ir(I)/squaramide cooperative catalysis.

The atroposelective azide−alkyne cycloaddition to offer axially chiral aryl triazoles was recently disclosed by Xu and co-workers via an Ir(I)/squaramide cooperative catalytic procedure (Scheme 4) [65]. It was found that the metal catalyst, reaction solvent, and temperature all play a key role in the increase in enantioselectivity. The cyclization process could be rationalized by the following mechanism: the crucial vinylidene ortho-quinone methide (VQM) **Int 9** could be formed from *1,5*-hydrogen migration triggered by the interaction between **10**, [Ir(COD)CI]_2_ and **L4**. The cycloaddition between chiral Ir(I) coordinated azide, and **Int 9** produced the chiral aryl triazole products. The **L4** serves both as an organic catalyst to activate ortho-hydroxy aryl alkyne **10** by hydrogen-bonding interaction between squaramide and oxygen atom in **Int 9**, and as a ligand to chelate with metal Ir. The aryl triazole skeleton bearing aryl, trimethylsilyl, heteroaryl, and alkyl substituent could also be achieved in excellent yield and high enantioselectivity with this protocol.

**Scheme 5:**
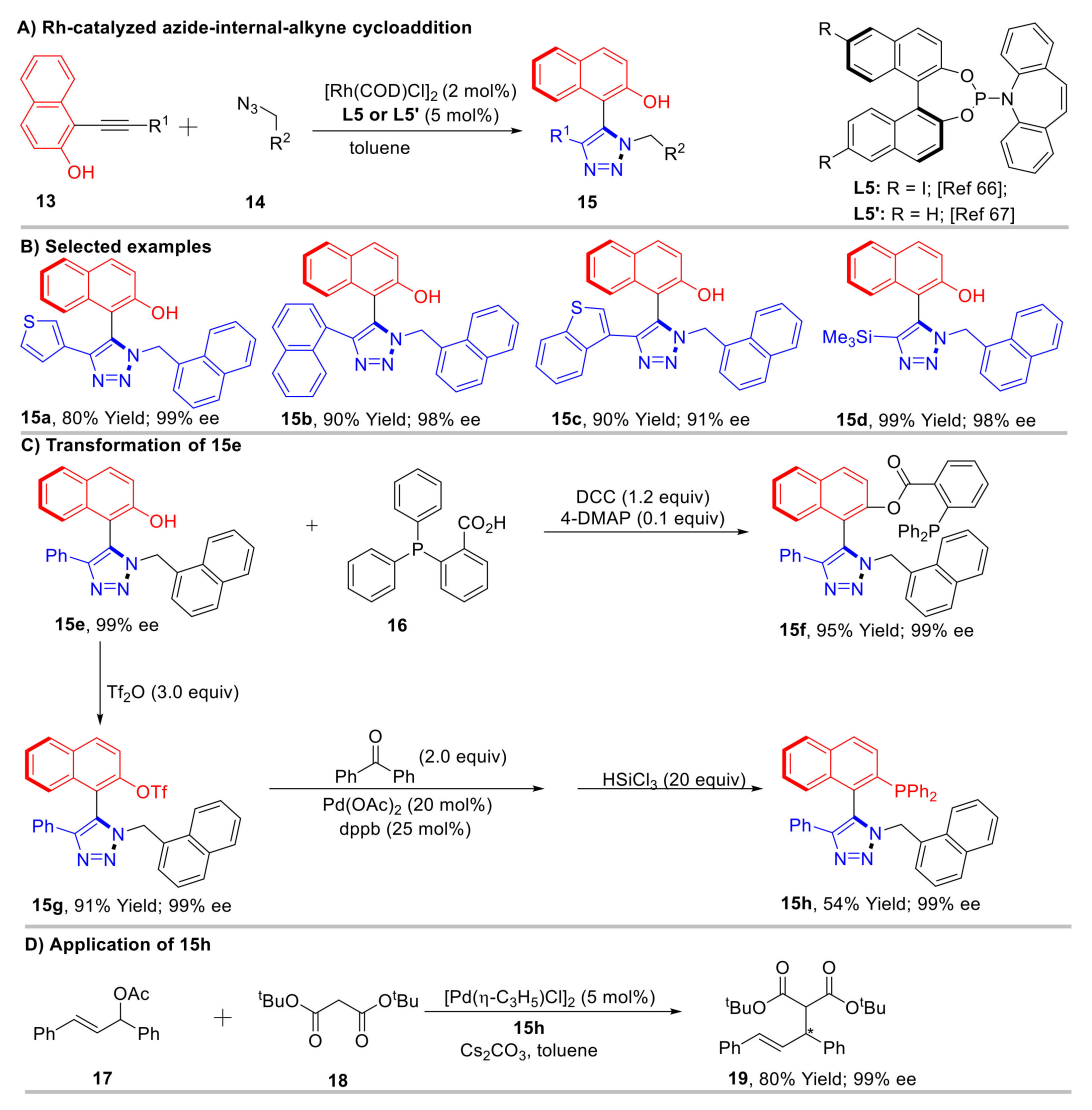
Rh-catalyzed azide-internal-alkyne cycloaddition.

At around the same time, structurally similar axially chiral 1,2,3-triazole derivatives were furnished by Li and Cui utilizing an Rh/chiral phosphoramidite ligand **L5** catalyst system through azide-internal-alkyne cycloaddition with high yield (up to 99%) and excellent enantioselectivities (up to 99% ee) (Scheme 5) [66,67]. Combining density functional theory calculations and control experiments, Li and co-workers presumed that the excellent regioselectivity of the catalytic system is closely related to the hydrogen-bond effect between the Cl-atom in [Rh(COD)Cl] and the H-atom in the hydroxyl group of phenol. Interestingly, in Li’s case, the utility of **15e** was also illustrated by converting it into new phosphine chiral ligands **15f** and **15h**. The latter compound (**15h**) was then employed as a chiral ligand for an asymmetric Tsuji–Trost allylation reaction to prepare chiral allylic alkylation product **19** in 99% ee.

#### 2.1.2. Enantioselective Synthesis of Fused Bicyclic Heteroaromatic Chiral Skeletons

**Scheme 6:**
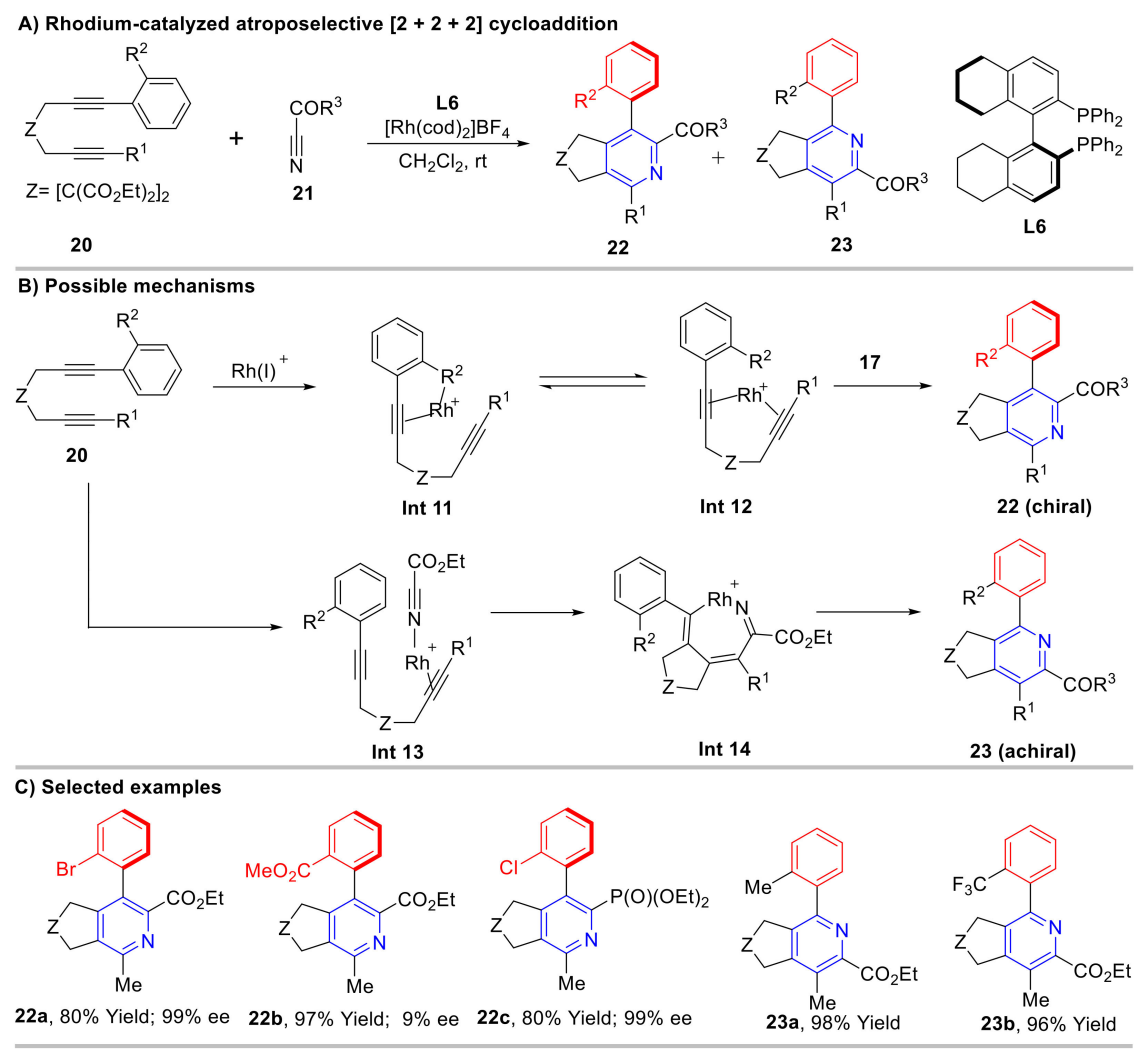
Rhodium-catalyzed atroposelective [2 + 2 + 2] cycloaddition.

The catalytic asymmetric Paal–Knorr reaction, heteroannulation, and azide−alkyne cycloaddition are effective strategies to produce atropisomers featuring pentatomic heteroaromatic molecules. Recently, the atroposelective [2 + 2 + 2] cycloaddition of nitriles **21** with (*o*-halophenyl) diynes **20** was successfully developed to access the heterobiaryl compounds **22** (chiral) and **23** (achiral) (Scheme 6) [68]. (*S*)-H_8_−BINAP **L6** was selected as the ligand to control the stereochemistry of the products. It was supposed that the equilibrium between intermediates **Int 11** and **Int 12** could be controlled by introducing the coordinating group (CO_2_Me, Cl, and OMe) at ortho-substituent (R^2^) to form **Int 12**. Oxidative cyclization of **Int 12** and insertion of nitrile **21** could give axially chiral product **22**. However, when the R^2^ was substituted by a less-coordinating group (such as CH_3_, etc.), the cationic rhodium(I) center could be coordinated by the alkyne moiety of **20** and nitrile of **21**, which was followed by oxidative cyclization, insertion of the pendant alkyne, and reductive elimination affording the achiral 6-arylpyridine **23**.

**Scheme 7:**
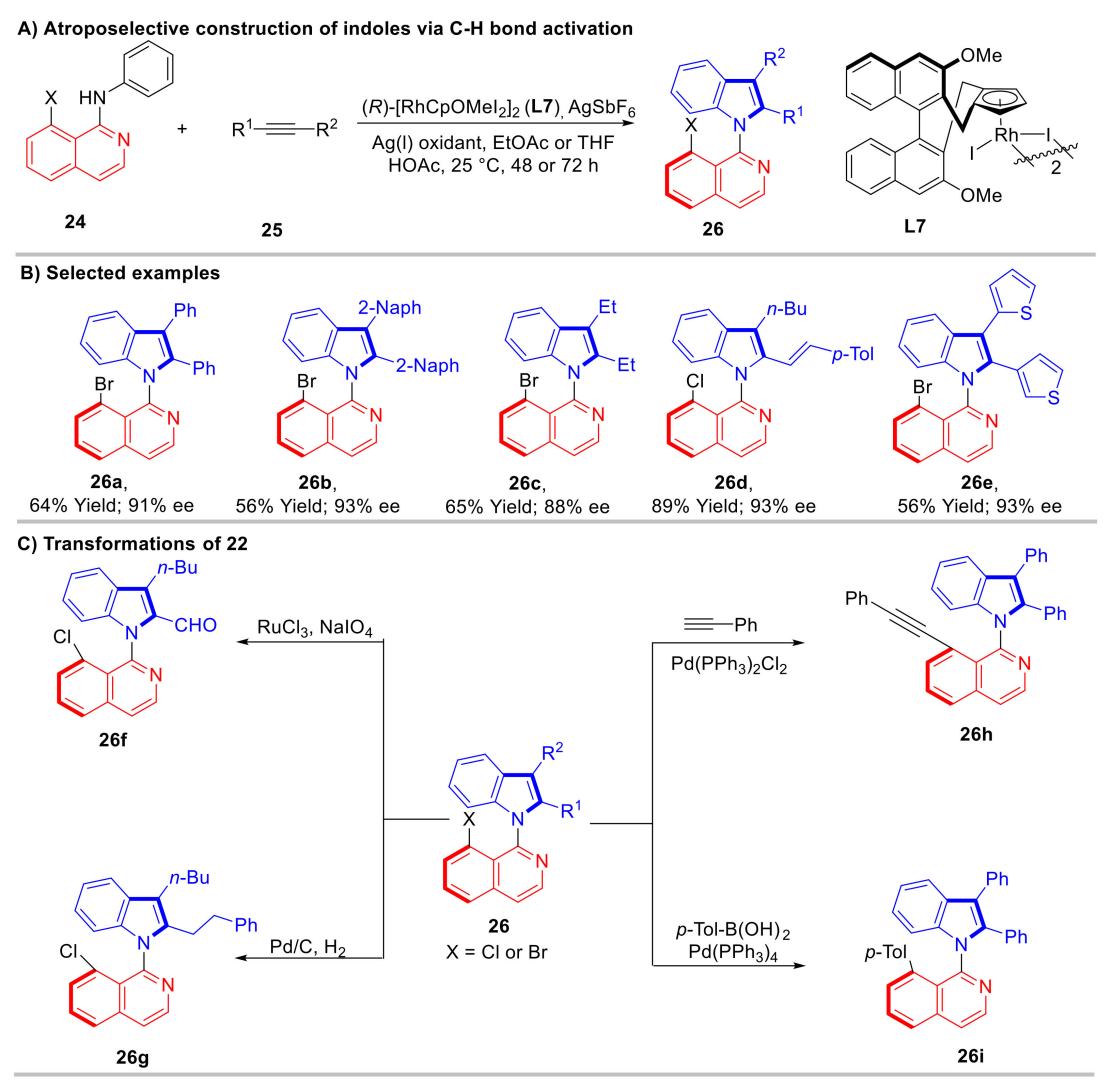
Atroposelective construction of indoles via C-H bond activation.

Inspired by the C-H activation approach to form heterocyclic compounds, Li and co-workers reported an innovative way to access isoquinolines bearing an indole sub-unit via rhodium (III)-catalyzed oxidative [3 + 2] annulation between internal alkynes **25** and aniline-substituted isoquinolines **24** (Scheme 7) [69]. Further study of the mechanism showed that the C-N reductive elimination and dynamic kinetic annulation were the decisive steps for controlling the stereo configuration. In addition, the *N*-isoquinolylindole **26** could be transformed into various derivatives (**26f**–**26i**) through oxidative C=C cleavage, reductive reaction, Sonogashira reaction, and Suzuki coupling.

**Scheme 8:**
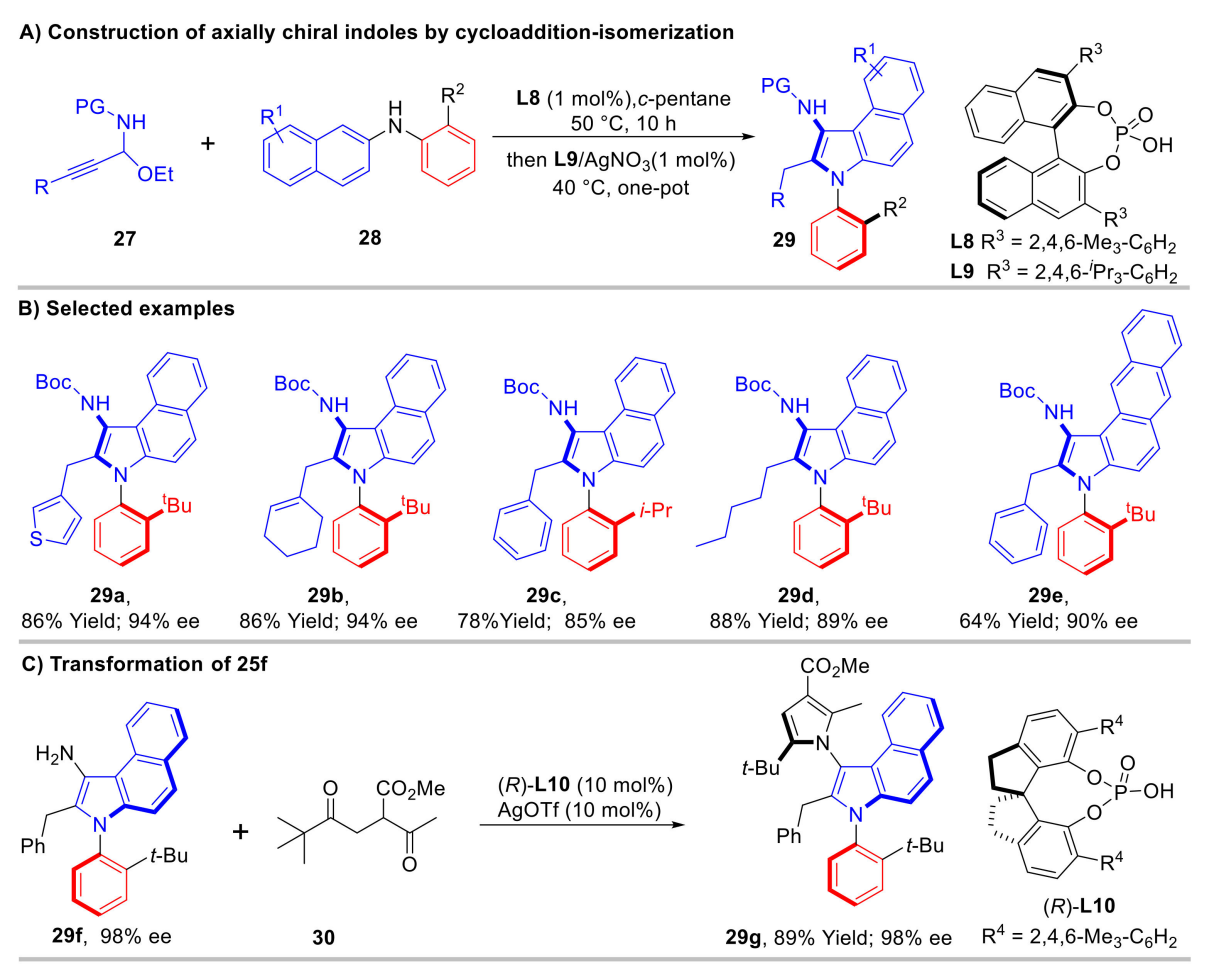
Construction of axially chiral indoles by cycloaddition–isomerization.

Recently, the de novo construction of indole-based frameworks has gained increasing attention since Kitagawa and co-workers disclosed the first example of axially chiral indoles [70]. Most reported methods focus on the mono-catalysis strategy to produce the heteroarene skeleton. In sharp contrast, there are rarely reports on the construction of axially chiral indoles via dual catalysis. In 2022, Shao’s group utilized organic catalysts (**L8** and the **L9**)/transition-metal (AgNO_3_) combined dual catalytic system for de novo synthesis of the axially chiral indole-based scaffold **29** with good yields and excellent enantioselectivities, by employing 2-naphthylamines **28** and N, O-acetals **27** as the starting materials (Scheme 8) [71]. This transformation offered a rare example of the combination of two chiral phosphoric acids with a metal catalyst in a single reaction to enable inaccessible molecules through intermolecular cycloaddition-isomerization. Impressively, **29g**, featuring two C-N axes, could be obtained by this method, which is challenging to access by existing strategies.

**Scheme 9:**
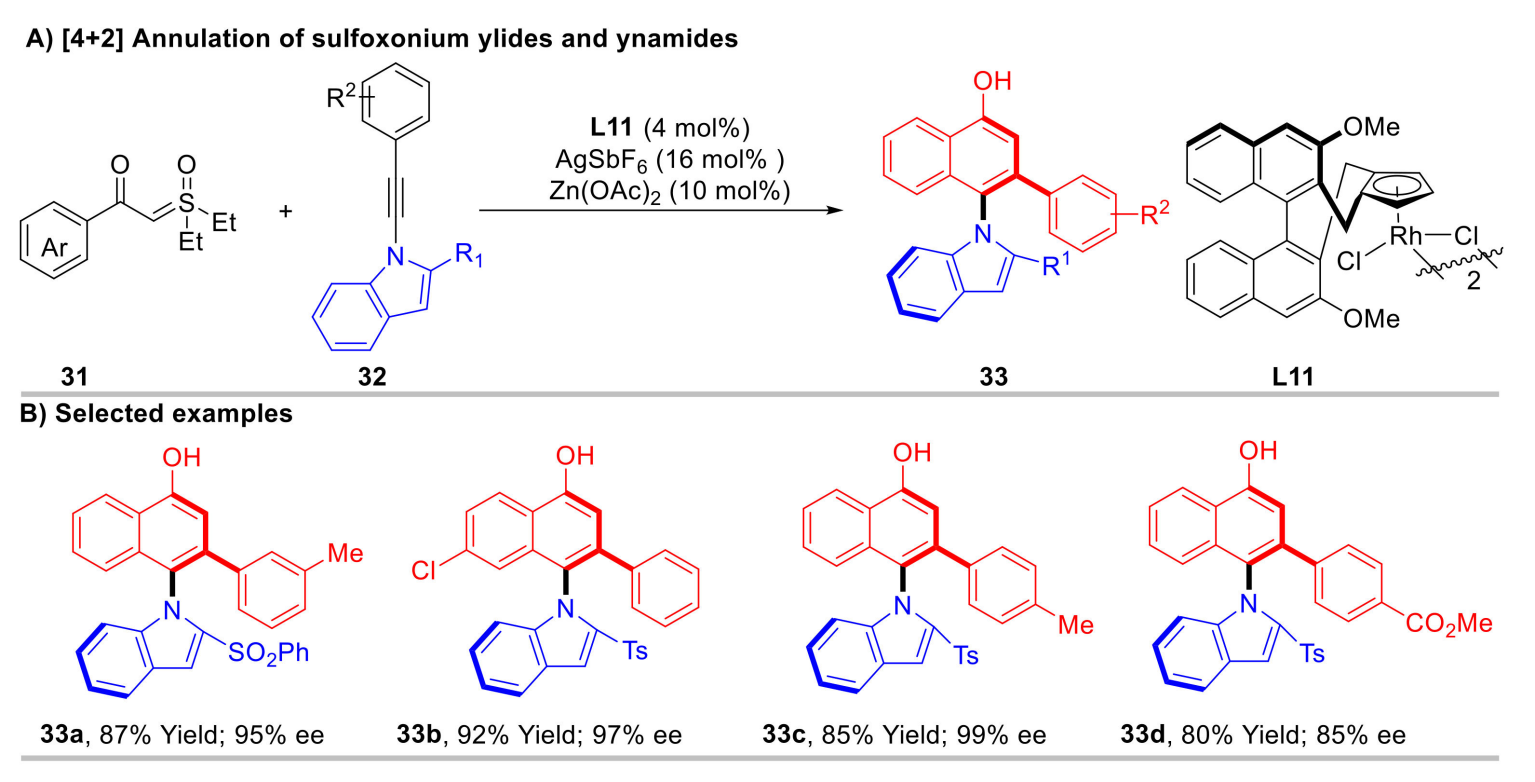
[4 + 2] Annulation of sulfoxonium ylides and ynamides.

Later, the asymmetric [4 + 2] annulative coupling reaction of arylalkynes **32** with different sulfonium ylides **31** mediated by **L11**, Zn(OAc)_2_, and AgSbF_6_ to furnish C-N axially chiral 4-indole 1-naphthols with wide substrate universality (**33a**–**33d**) was also established by Li’s group (Scheme 9) [72]. Furthermore, this report indicated that sulfonium ylides **31** could be applied as a versatile platform in the asymmetric C-H bond activation process.

Later, the asymmetric [4 + 2] annulative coupling reaction of arylalkynes **32** with different sulfonium ylides **31** mediated by **L11**, Zn(OAc)_2_, and AgSbF_6_ to furnish C-N axially chiral 4-indole 1-naphthols with wide substrate universality (**33a**–**33d**) was also established by Li’s group (Scheme 9) [72]. Furthermore, this report indicated that sulfonium ylides **31** could be applied as a versatile platform in the asymmetric C−H bond activation process.

### 2.2. Intramolecular Cyclization Reaction Catalyzed by Transition Metal Catalysts

#### Enantioselective Synthesis of Heteroaromatic Chiral Skeleton with Fused Ring

**Scheme 10:**
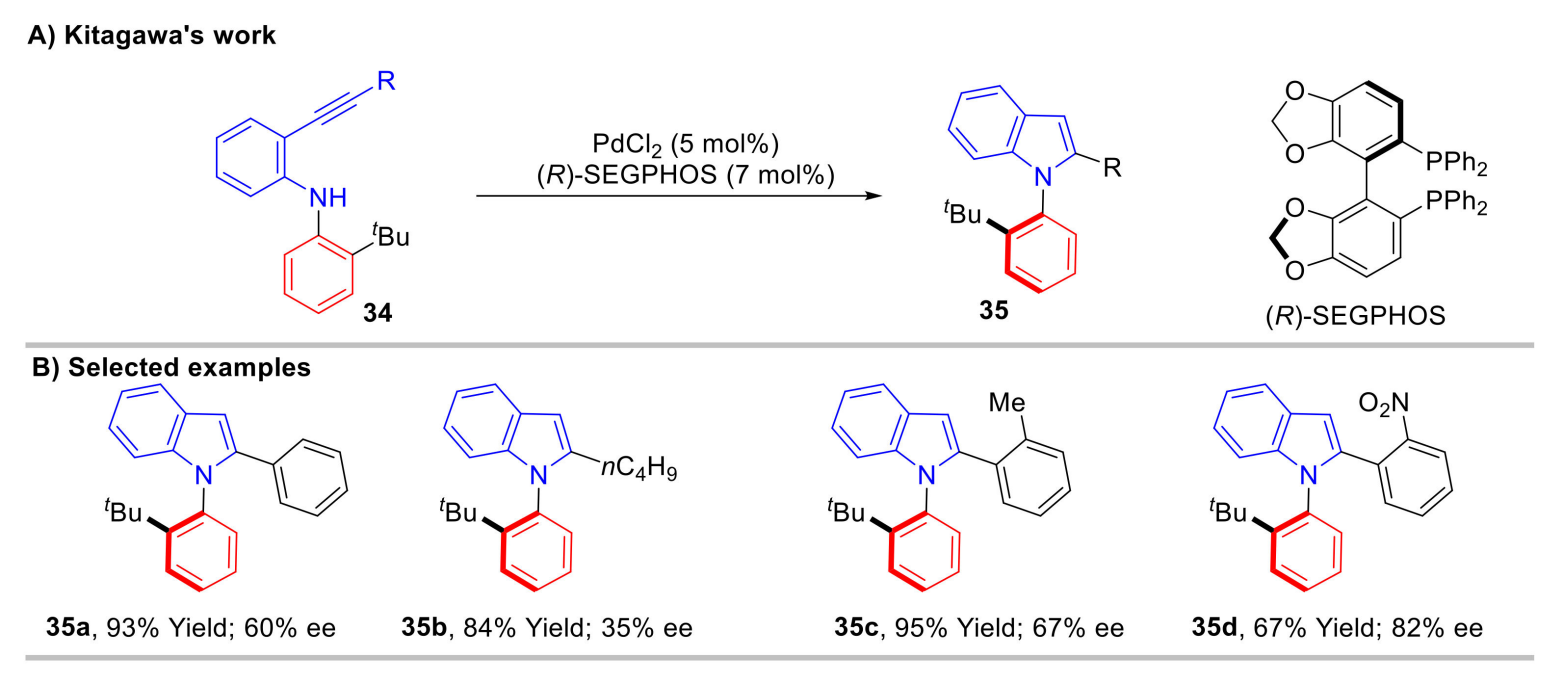
Atropisomeric indoles with an N-C chiral axis.

Intramolecular cyclization reactions catalyzed by transition metal catalysts are considered powerful methods for delivering a variety of atropisomeric indoles with a chiral axis. In 2010, *N*-(ortho-tert-butylphenyl)-2-(phenylethynyl) aniline **34** was used to construct indole compounds with N-C axial chirality through intramolecular hydroamino cyclization catalyzed by PdCl_2_ in the presence of (*R*)-SEGPHOS by Kitagawa and co-workers (Scheme 10) [70].

**Scheme 11:**
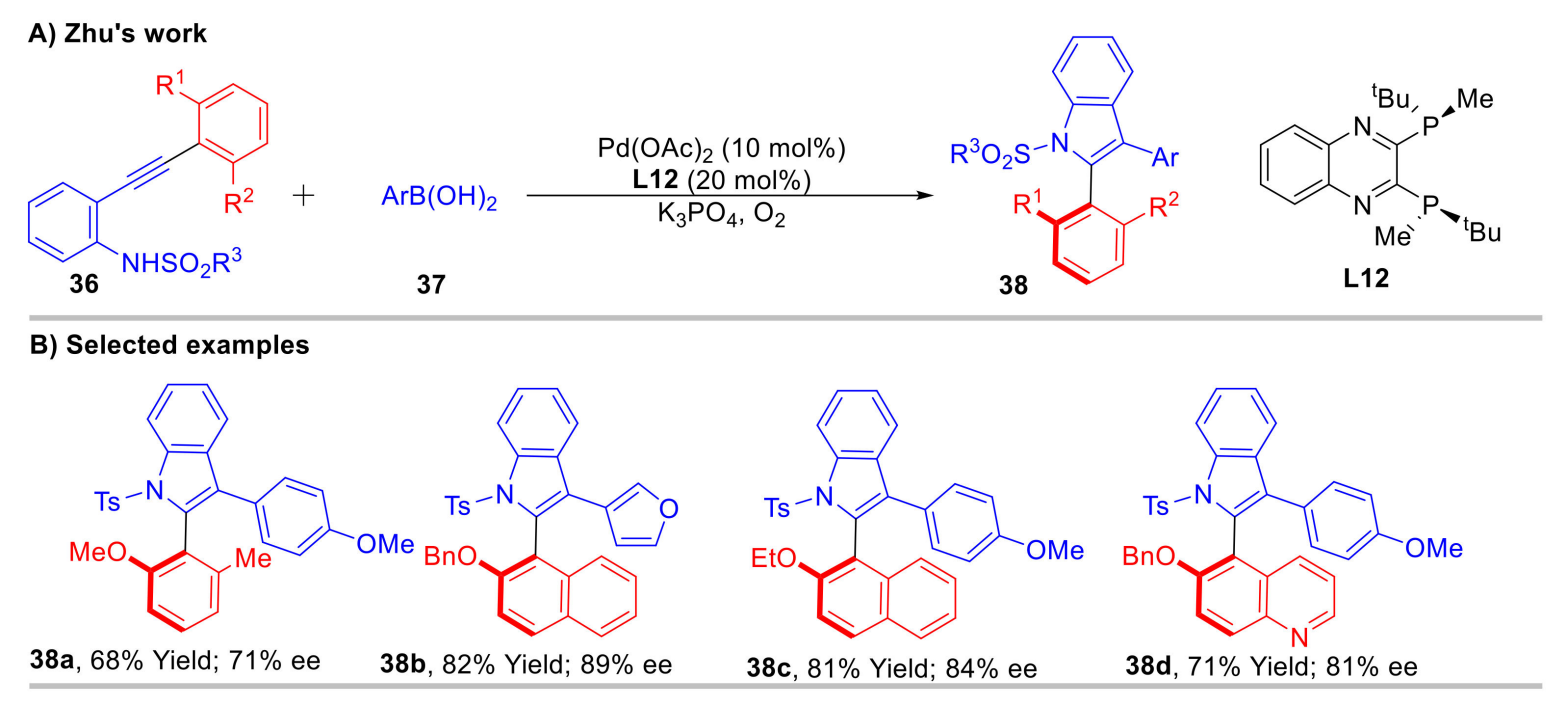
Palladium-catalyzed enantioselective Cacchi reaction.

**Scheme 12:**
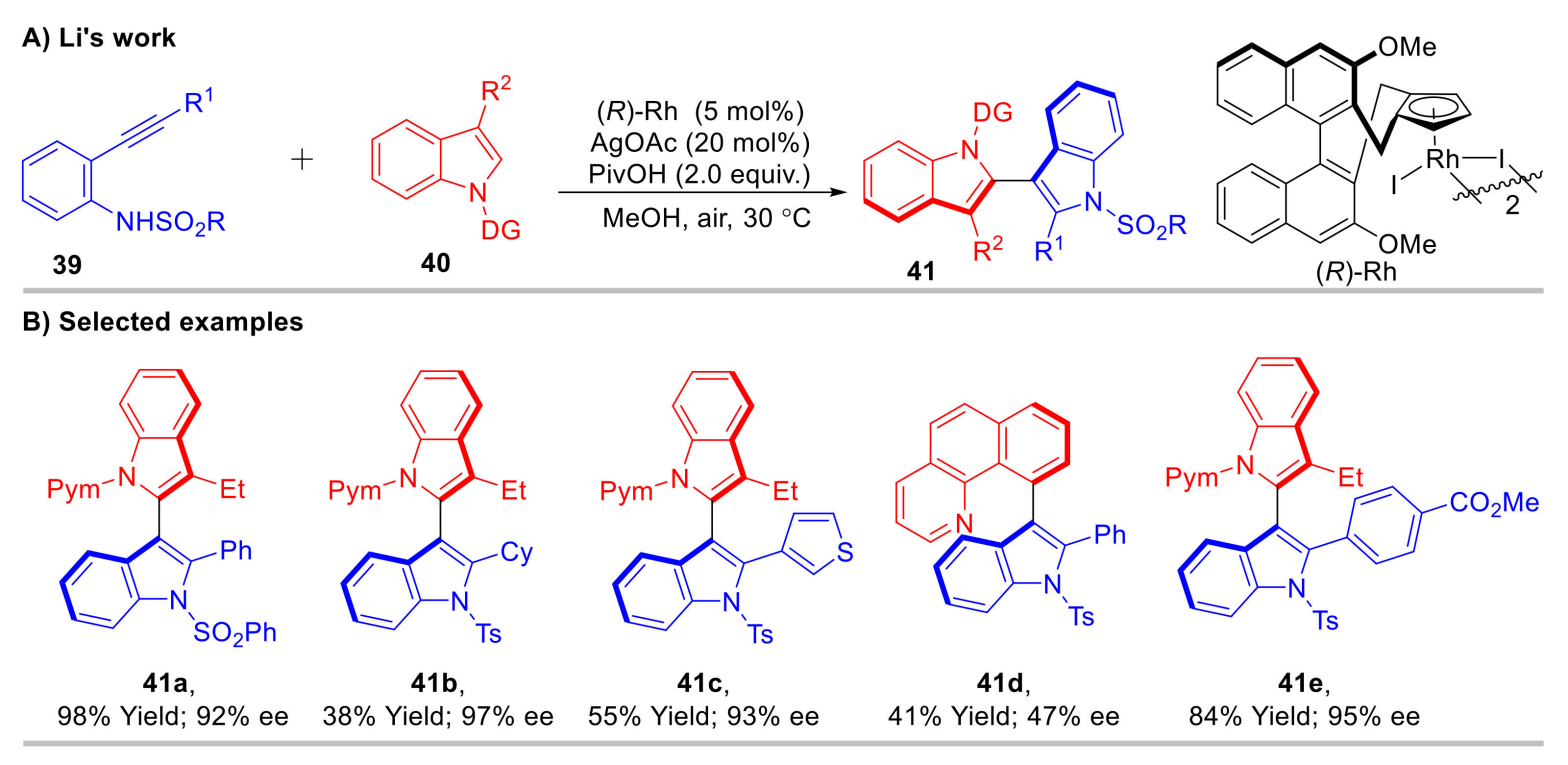
Rh(III)-catalyzed asymmetric synthesis of biindolyls.

**Scheme 13:**
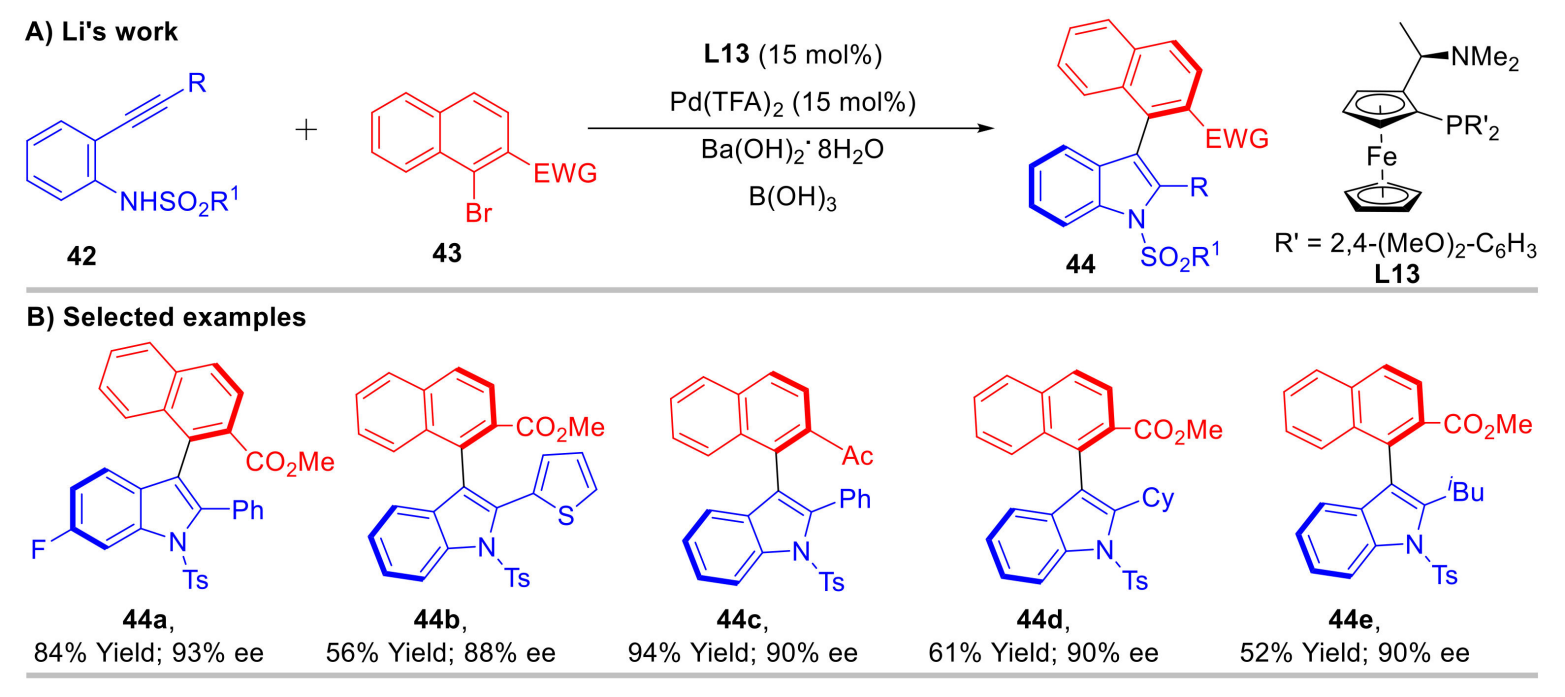
Construction of atropisomeric 3-arylindoles.

The above work opened a new field of the catalytic asymmetric synthesis of axially chiral indole-based framework utilizing *o*rtho-alkynylaniline. For example, in 2020, Zhu’s group revealed the strategy of enantioselective Cacchi reaction through a Pd(OAc)_2_/(R, R)-QuinoxP* **L12** catalyzed cascade process to provide enantioenriched 2,3-disubstituted indoles **38** possessing a chiral C2-aryl axis with excellent enantioselectivities (Scheme 11) [73]. Furthermore, the newly formed indole ring possessed unique properties, for example, high aromaticity and a planar 2D structure, and was electronically rich which enables it to be a good hydrogen-bond donor that can be easily functionalized. Therefore, based on these inherent characteristics, Li and co-workers achieved axially chiral biindolyls using a unified chiral Rh (III) catalyst through intermolecular C-H activation followed by intramolecular alkyne cyclization (Scheme 12) [74]. Unlike other reported methodologies, this conversion does not require an inorganic base as an additive and achieved the enantiomeric 2,3′-biindolyls under very mild reaction conditions with excellent enantioselectivity. Later, the same group performed the de novo construction of 3-arylindoles via an enantioselective Cacchi reaction between aryl bromides **43** and *o*-alkynylanilines **42** in the presence of Pd(TFA)_2_, ferrocene-based *N*, *P*-ligands **L13** under the assistance of Ba(OH)_2_ 8H_2_O and B(OH)_3_ (Scheme 13) [75].

**Scheme 14:**
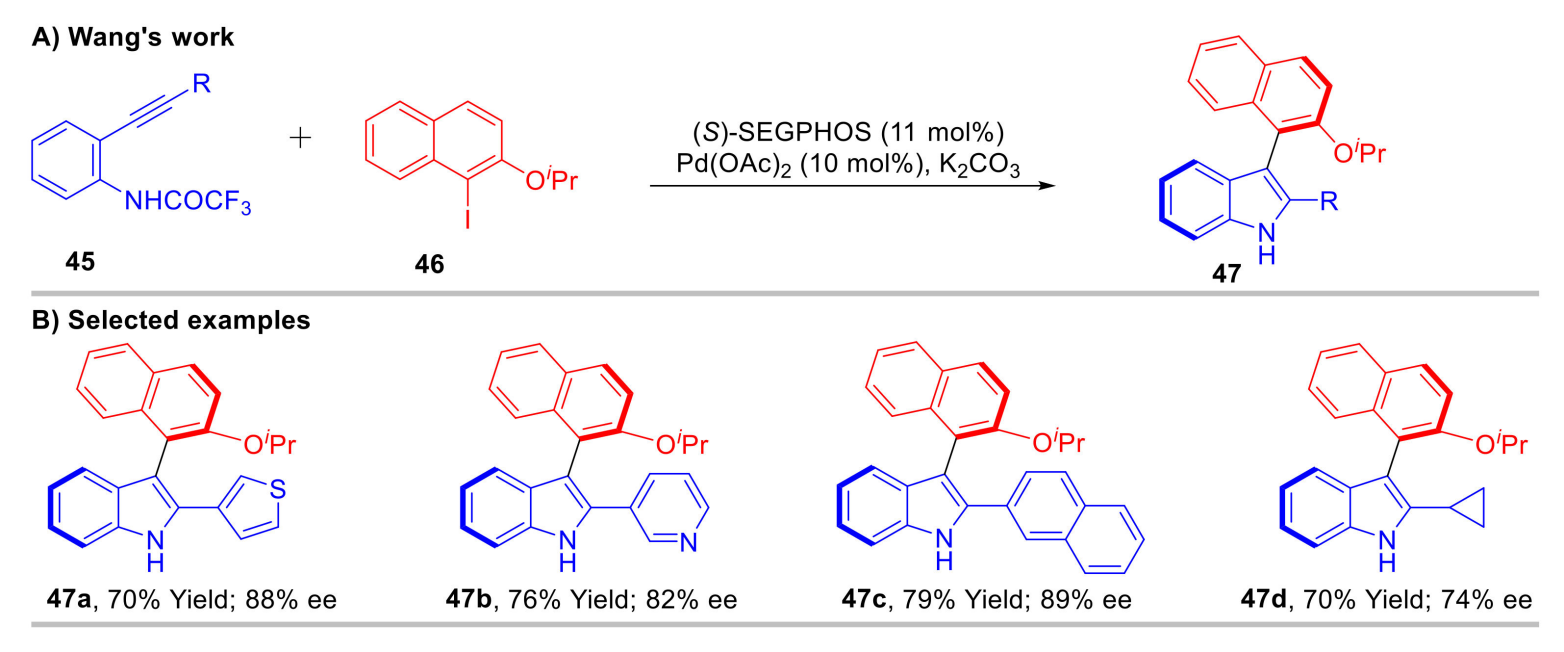
Asymmetric synthesis of naphthyl-C3-indoles.

**Scheme 15:**
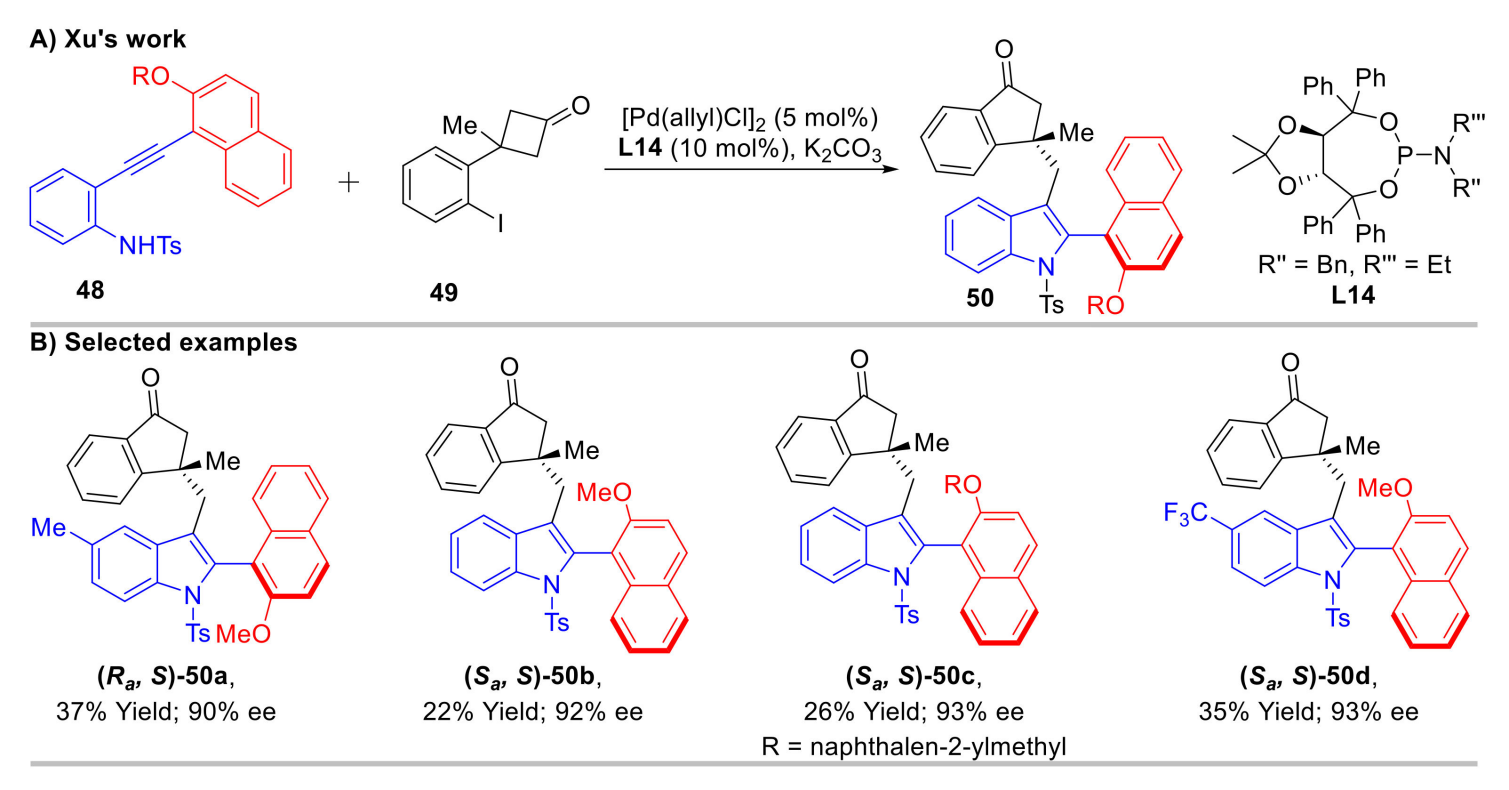
Pd-catalyzed enantioselective tandem C-C bond activation/Cacchi reaction.

Similar work was disclosed in the same year, in which 2-phenylethynyltrifluoroacetanilide **45** and 1-iodo-2-isopropoxynaphthalene **46** were used by Wang’s group as the reaction substrates for directly accessing naphthyl-C3-indoles with a free NH moiety (Scheme 14) [76]. Remarkably, it offered a strategy for improving the enantioselectivity by adjusting the steric hindrance of the substituted group on the O-atom of 1-iodonaphthalen-2-ol. In addition, it was found that dissolving the inorganic base in H_2_O could facilitate the formation of product by offering a homogeneous catalytic system. In 2021, Xu’s group also reported a cyclization reaction between o-ethynylanilines **48** and cyclobutanones **49** catalyzed by [Pd(allyl)Cl] in the presence of a chair phosphoramidite ligand **L14** to access indanone-substituted indole **50** with concomitant formation of C-N bond, C-C bond, and an all-carbon quaternary stereocenter (Scheme 15) [77].

**Scheme 16:**
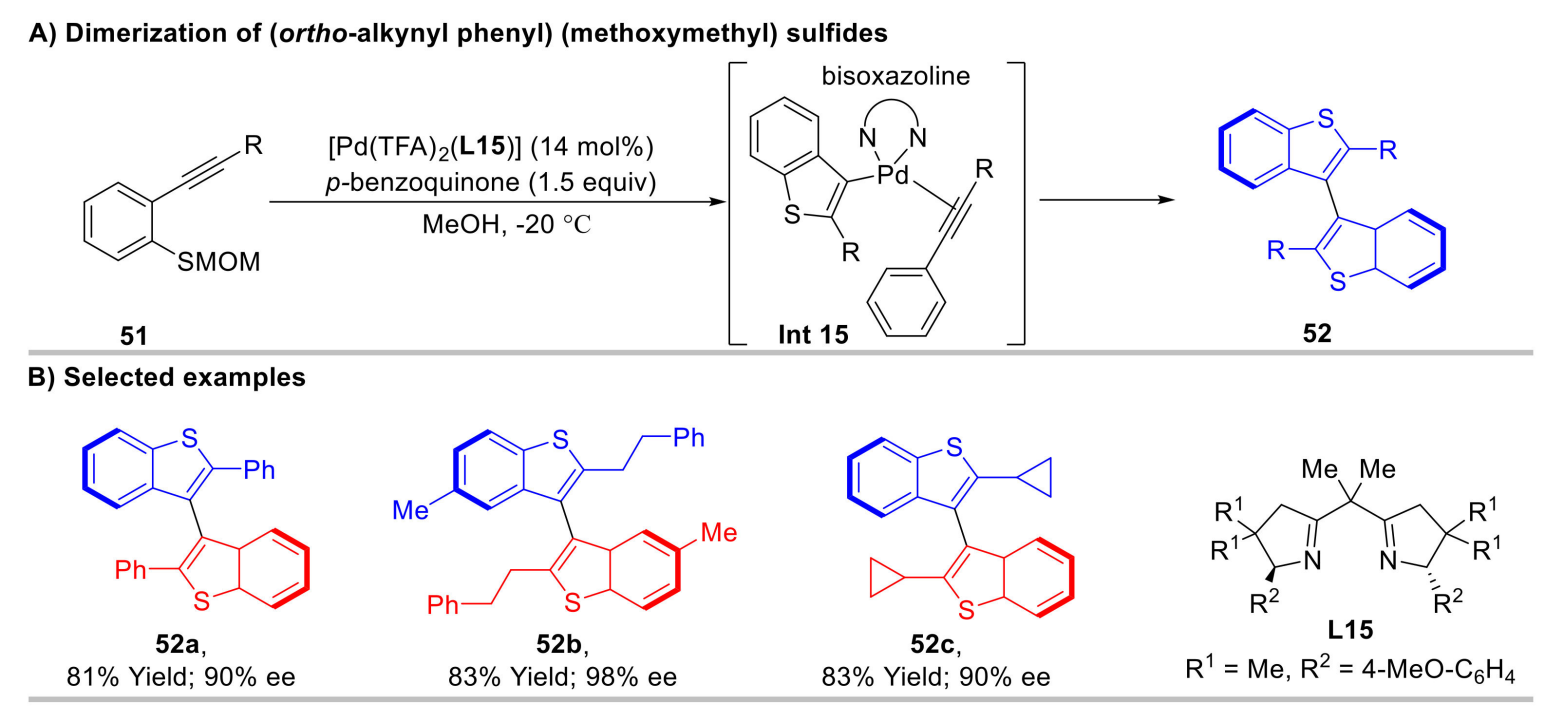
Dimerization of (ortho-alkynyl phenyl) (methoxymethyl) sulfides.

Methoxymethyl sulfides **51** were also employed to construct the axially chiral bibenzothiophenes **52** through asymmetric cyclization–dimerization (Scheme 16) [78]. The alkynophilicity of **Int 15** was enhanced by the box ligand **L15**, which promotes the occurrence of dimerization affording **52** with broad functional group tolerance.

**Scheme 17:**
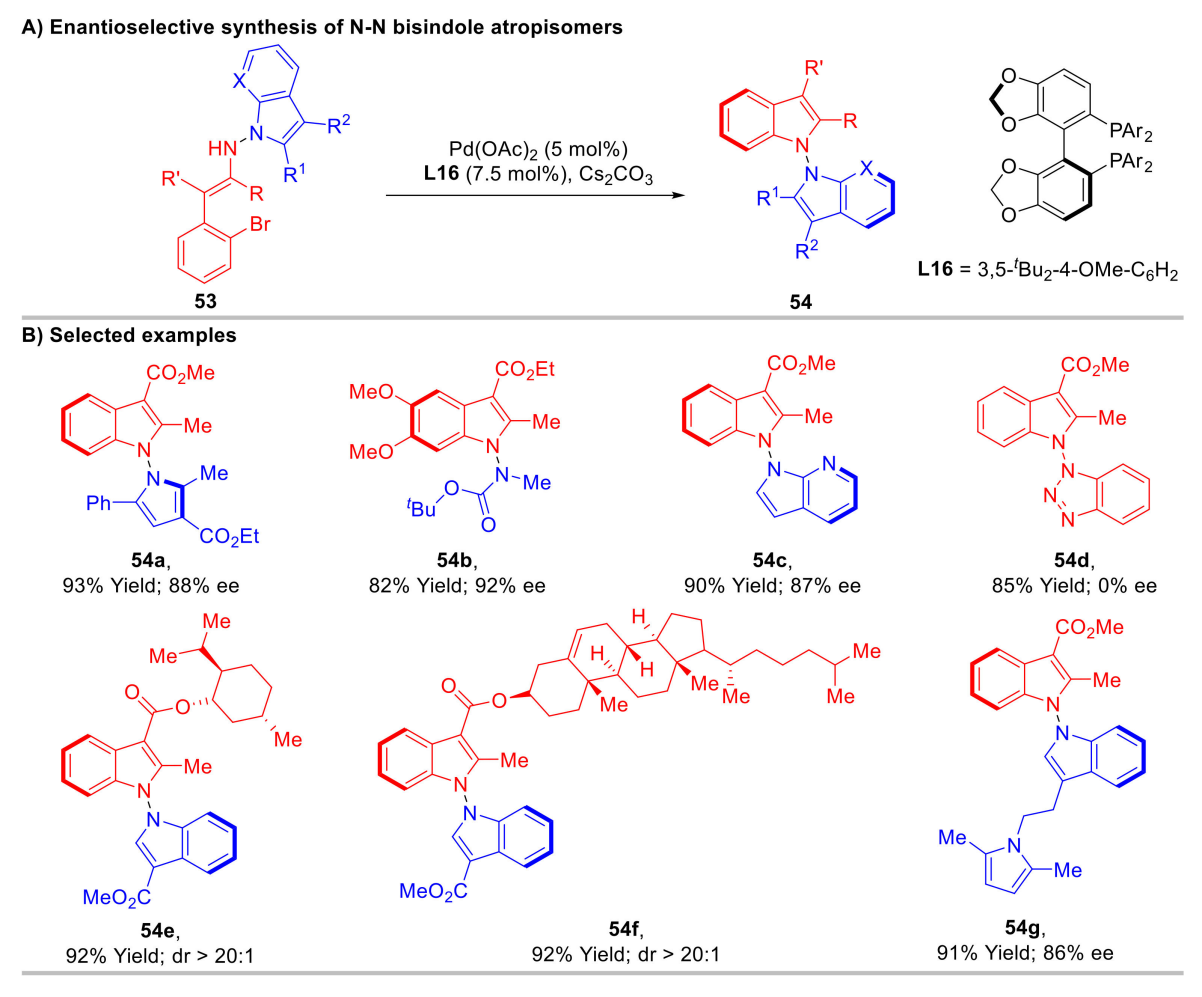
Enantioselective synthesis of *N*-*N* bisindole atropisomers.

Compared with these achievements for the synthesis of C-C and N-C axially chiral compounds, the methodology for the synthesis of N-N atropisomers was still in its infancy. In 2022, structurally diverse indole-heterocycle (carbazole and pyrrole) bearing a chiral N-N axis was accessed via intramolecular arylation of enamine **53** catalyzed by Pd(OAc)_2_ and DTBM-SEGPHOS (**L16**) (Scheme 17) [79]. Several compounds containing a core structure of active molecules were compatible with this protocol. Generally, the reaction delivered the corresponding product with high diastereoselectivities (**54e**–**54g**). However, because of the reduced steric hindrance, the similar indole/benzo[*d*] [1,2,3]-triazole atropisomer **54d** was provided as a racemic product.

In this section, a series of heterobiaryl frameworks have been provided with excellent yield and ee values catalyzed by (Rh, Ag, Ir, Pd, and Fe) with the assistance of chiral phosphoric acid ligand, phosphine ligand, and chiral rhodium (III) cyclopentadienyls. Significantly, chiral phosphoric acid can be activated by a Lewis acid to promote many challenging transformations. In addition, pentatomic heteroaromatics bearing pyrrole, 1,2,3-triazole could be accessed by the classical reaction. However, the synthesis of oxygen and sulfur-containing heterobiaryl catalyzed by transition metal catalysts was rarely developed.

## 3. Synthesis of Axially Chiral Heterobiaryl Scaffold Catalyzed by Organocatalysts through De Novo Synthesis

Asymmetric organocatalysis has been employed as a powerful tool to furnish chiral products in an enantioenriched form. To date, a variety of organocatalyst-catalyzed synthetic approaches have been reported for constructing axially chiral heterobiaryl molecules via new ring formation. These tactics could be divided into two major themes: (1) Intermolecular cyclization reactions catalyzed by organocatalysts; (2) Intramolecular cyclization reactions catalyzed by organocatalysts.

### 3.1. Intermolecular Cyclization Reaction catalyzed by Organocatalysts

#### 3.1.1. Enantioselective Synthesis of Heteroaromatic Chiral Skeleton with a Five-Membered Ring

**Scheme 18:**
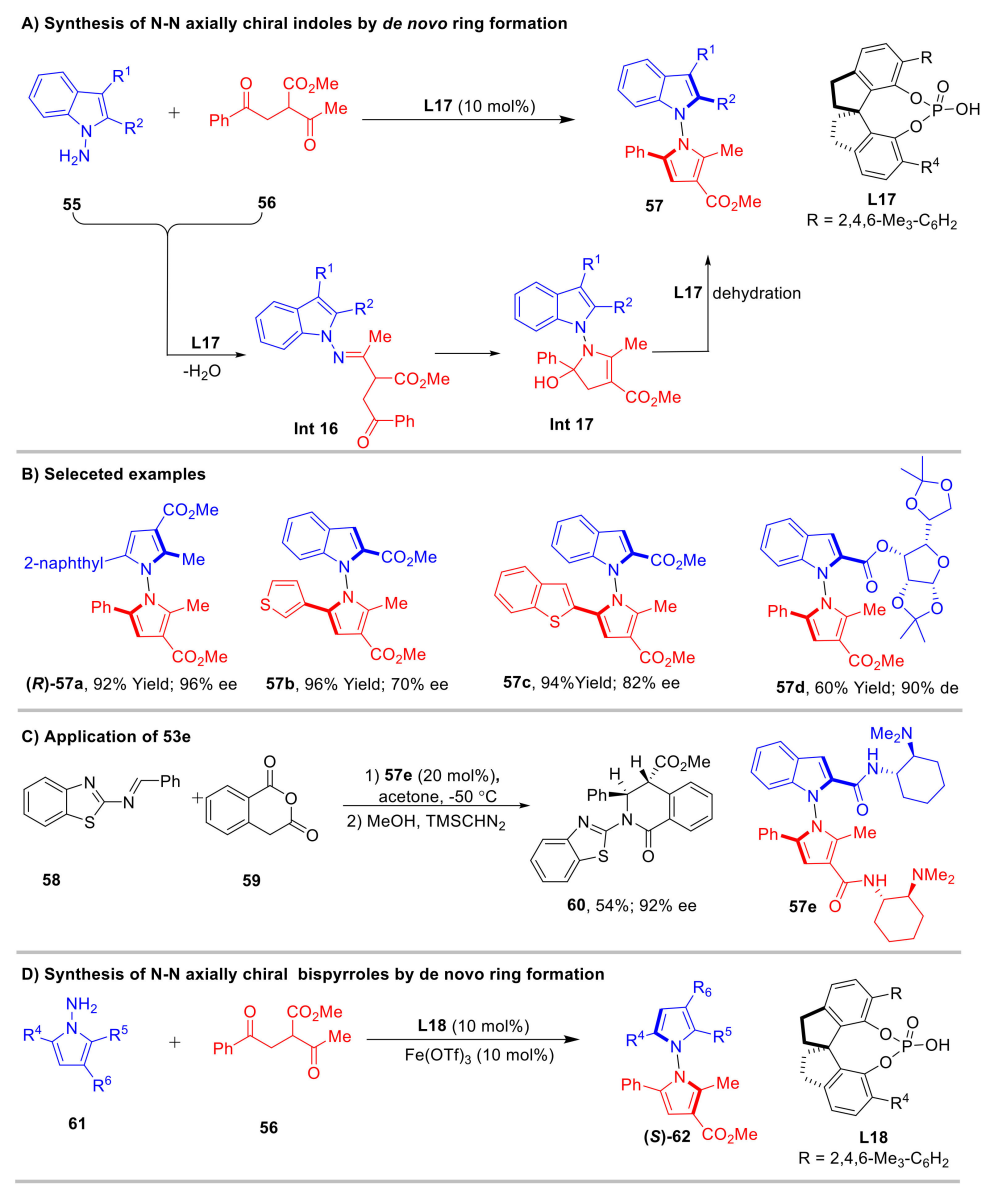
Synthesis of *N*-*N* axially chiral indoles by de novo ring formation.

In 2022, Shi and co-workers established a chiral phosphoric acid catalyzed Paal–Knorr reaction of *N*-aminoindole **55** with 1,4-diketones **56** affording N-N axially chiral indoles and pyrroles (Scheme 18) [80]. The stereoselectivity of this transformation was generated by the bulky substituted group at the C2-position of *N*-aminoindoles or *N*-aminopyrroles, which restricted the free rotation of the *N*-*N* axis. The free amino group of **55** underwent an asymmetric annulation reaction with 1,4-diketone **56** catalyzed by chiral phosphoric acid to produce intermediate **Int 16**. **Int 16** then cyclized intramolecularly affording **Int 17.** Dehydration of **Int 17** gave the final axially chiral product **57**. Furthermore, the methodology could also be extended to the synthesis of axially chiral 1,1′-bipyrrole **57a**. The preliminary application of N-N axially chiral bispyrroles as organocatalysts was also demonstrated by a [4 + 2] cycloaddition between **58** and **59** to provide chiral product **60** in 92% ee. At the same time, Zhao’s group disclosed a chiral phosphoric acid catalyzed Paal–Knorr reaction of *N*-aminopyrroles **61** with 1,4-diketones **56** to access 1,1′-bipyrroles **62**. Interestingly, it was found that Fe(OTf)_3_ could induce enantiodivergence, as in the presence of Fe(OTf)_3,_ the reaction delivered enantiomeric (*S*)-**62**, while the same reaction without Fe(OTf)_3_ produced its enantiomer (*R*)-**62** [81].

**Scheme 19:**
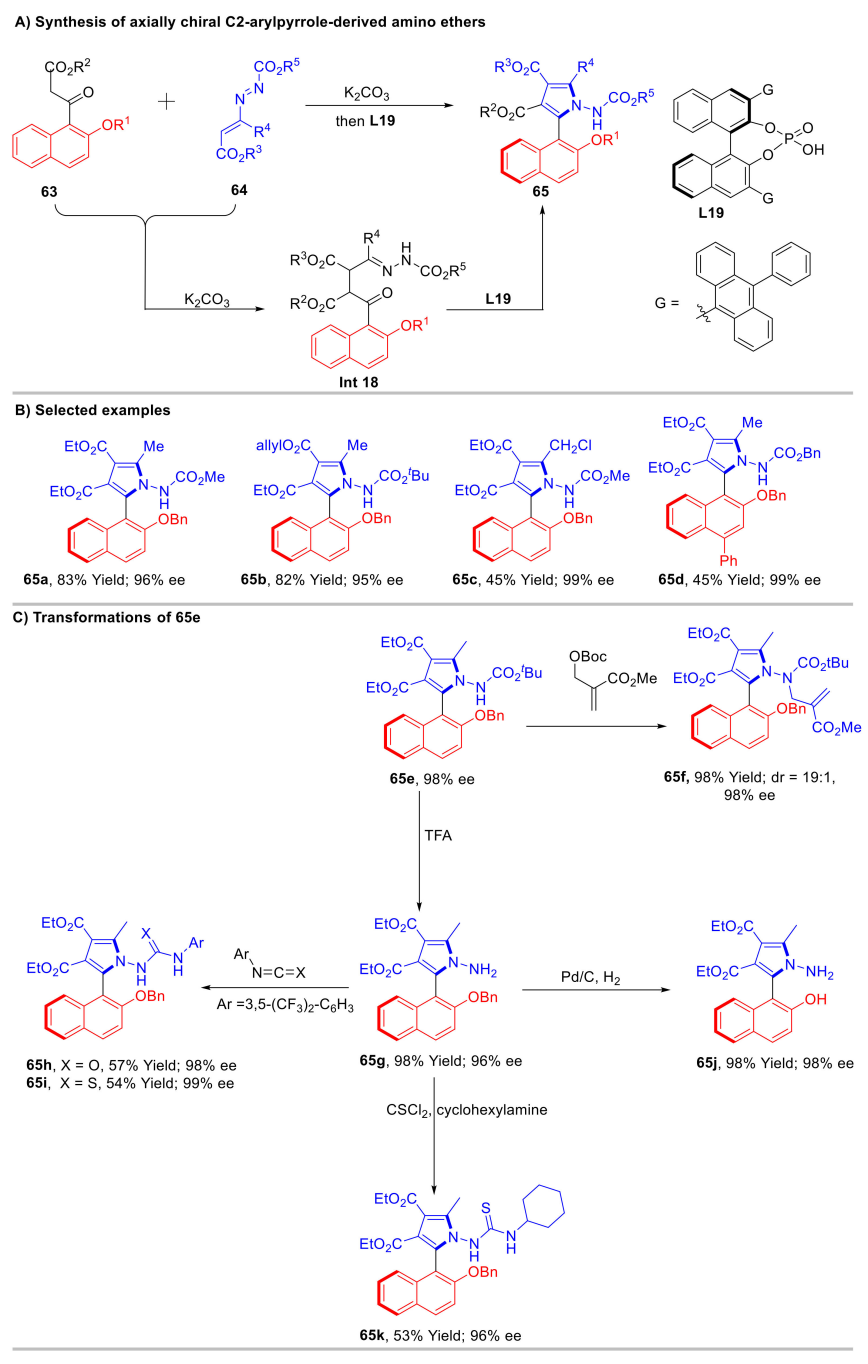
Synthesis of axially chiral C2-arylpyrrole-derived amino ethers.

Shortly after this, Mei’s group reported a procedure to construct C1-symmetric biaryl amino ethers (a privileged chiral backbone) via an asymmetric Attanasi reaction of azoalkenes **64** with 1,3-dicarbonyl compound **63** catalyzed by chiral phosphoric acid **L19** (Scheme 19) [82]. In addition to the synthesis of a range of axially chiral biaryl amino ethers, various further conversions starting from **65e** were also carried out. For example, **65f** bearing biaryl C-C axis and N-N axis was produced by N-allylic alkylation of **65e**. Deprotection of the Boc group in **65e** afforded **65g** with a free amino group in excellent yield. Thioureas derivatives **65h**, **65i**, **65k** could be obtained with retention of the enantioselectivities from **65g**. In addition, the hydrogenation of **65g** could also provide the parent NPNOL (1-(1-aminopyrrol-2-yl) naphthalen-2-ol) scaffold **65j** in excellent yield and enantioselectivity.

#### 3.1.2. Enantioselective Synthesis of Heteroaromatic Chiral Skeleton with Fused Ring

**Scheme 20:**
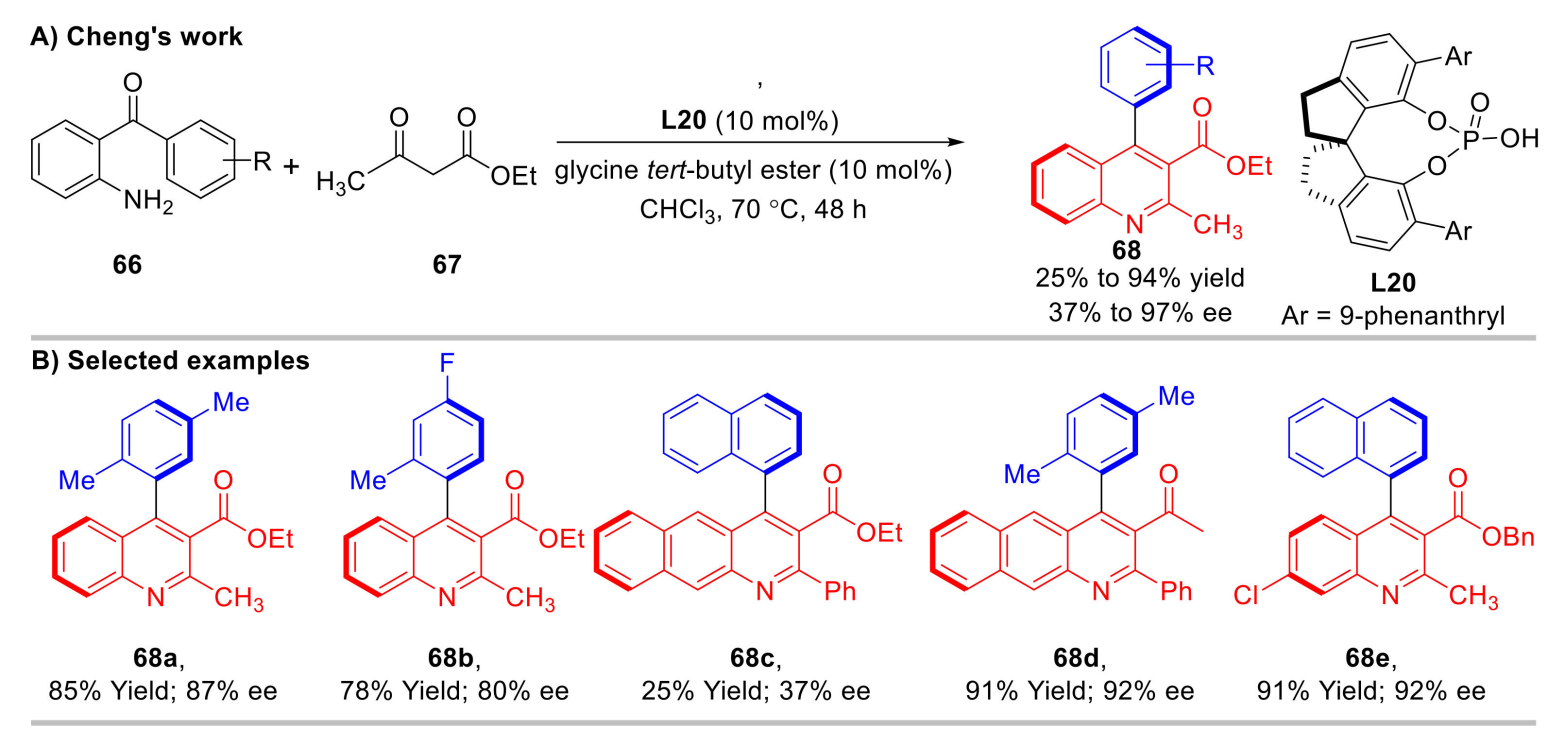
Organocatalytic atroposelective Friedländer quinoline heteroannulation.

**Scheme 21:**
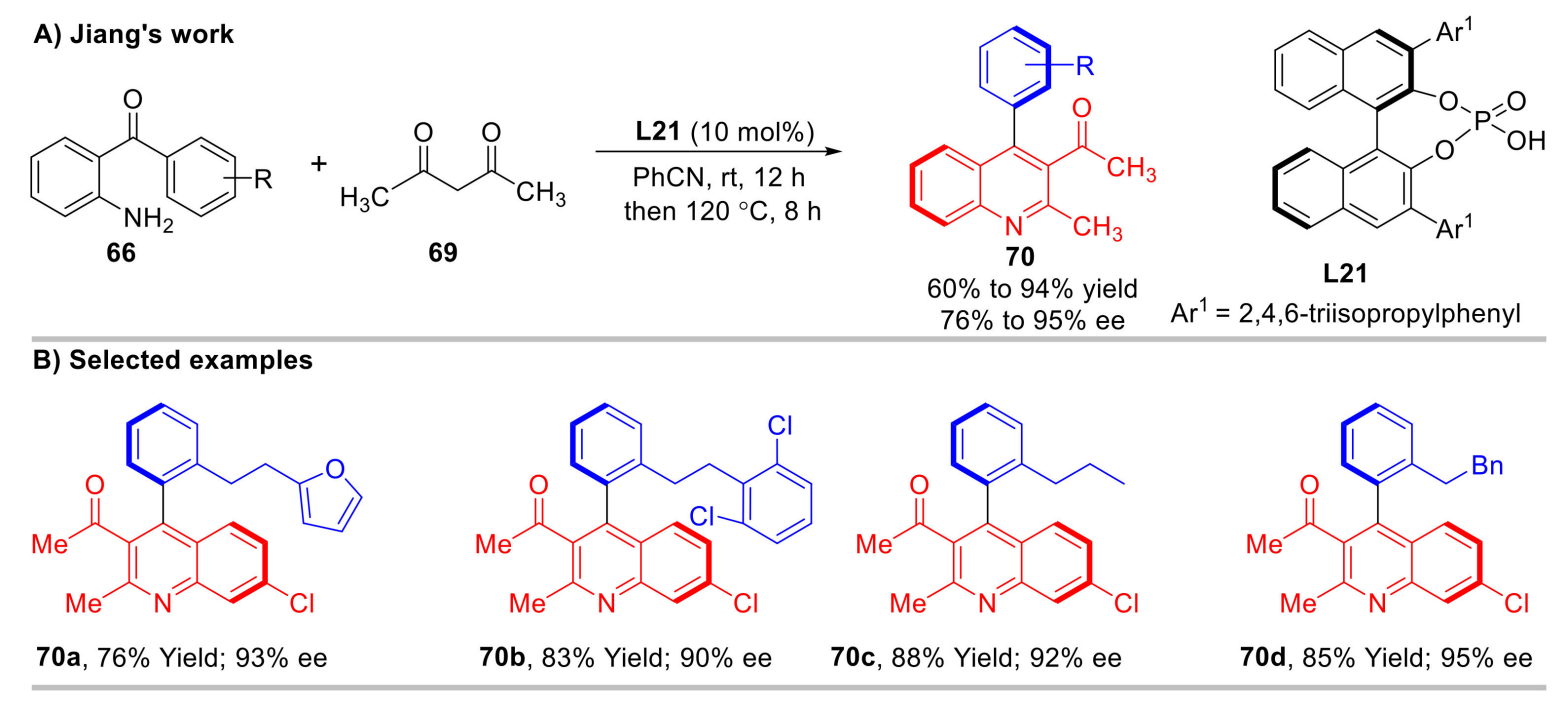
Asymmetric synthesis of atropisomeric quinolines.

**Scheme 22:**
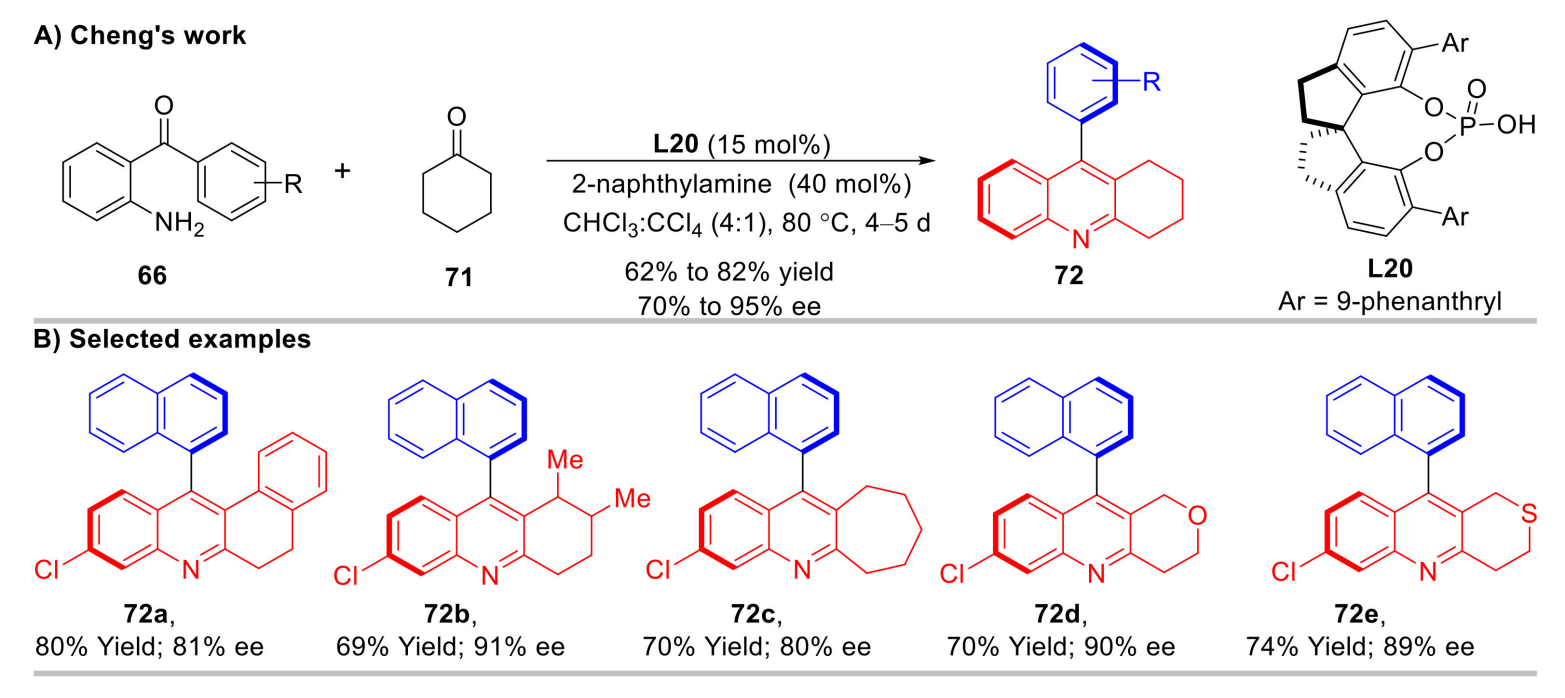
Diastereoselective synthesis of 9-aryltetrahydroacridines.

**Scheme 23:**
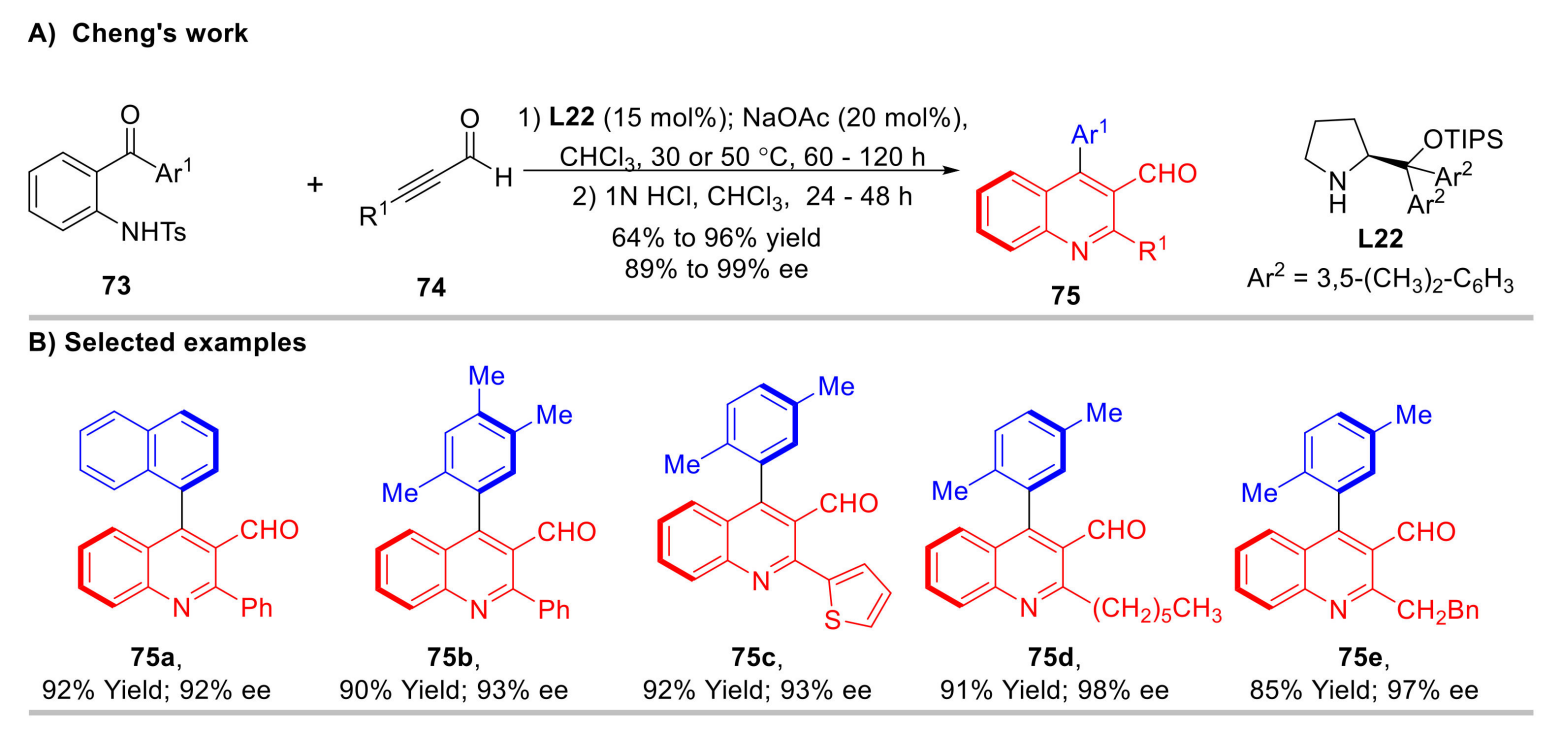
Atroposelective construction of 4-naphthylquinoline-3-carbaldehydes.

**Scheme 24:**
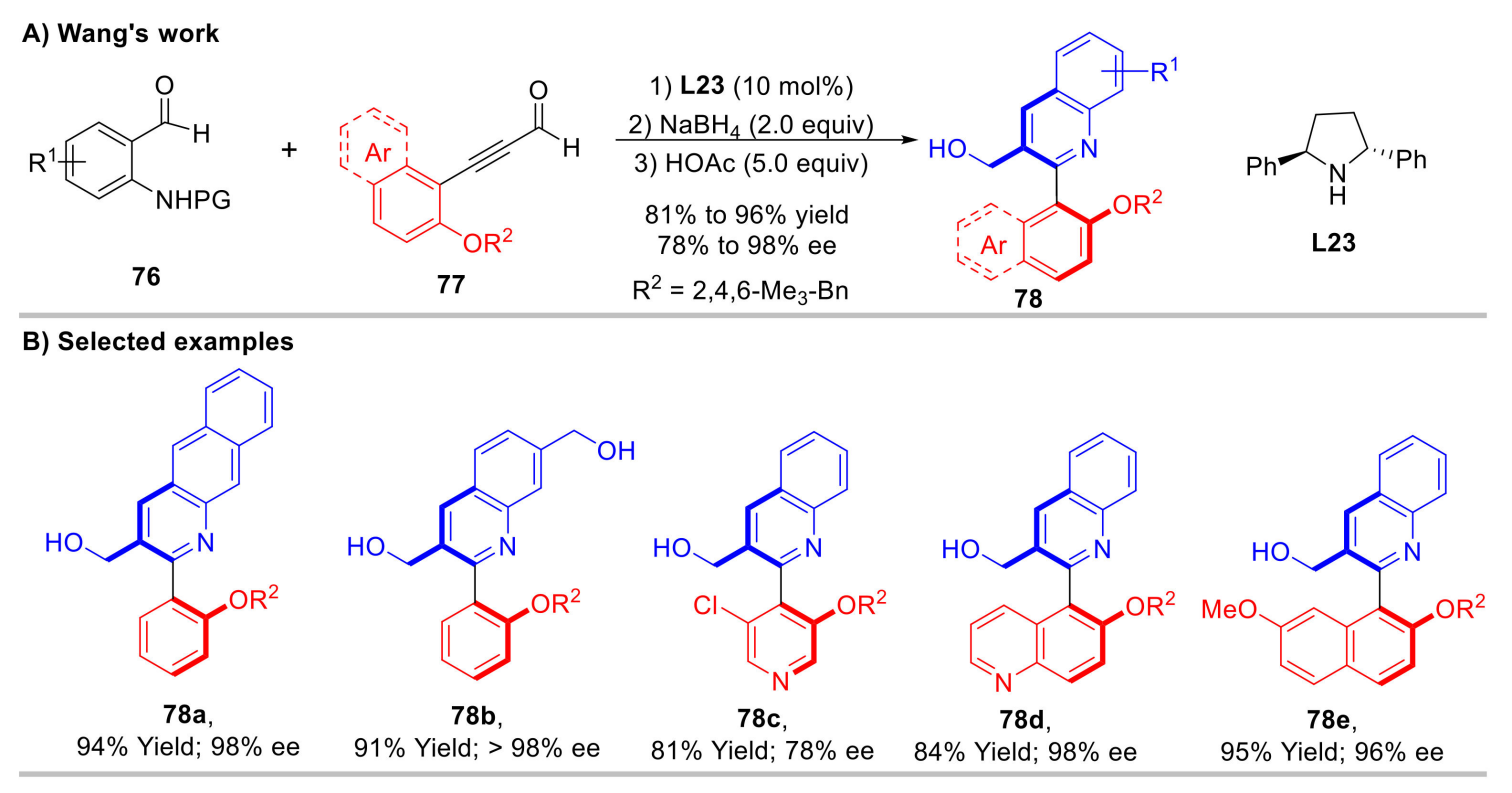
The synthesis of axially chiral 2-arylquinolines.

Since quinoline derivatives exist widely in pharmacological molecules and natural products, many synthetic strategies have been developed for their construction. Recently, Jiang’s and Cheng’s group uncovered successively effective protocols to produce atropisomeric quinoline scaffold **68**, **70**, and **72** through a Friedländer reaction between 2-aminoaryl ketones **66** and ketone derivatives (ethyl acetoacetate **67**, acetylacetone **69** and cyclohexanone **71**) catalyzed by chiral phosphoric acids **L20** and **L21** in good yields with high enantioselectivities (Scheme 20, Scheme 21 and Scheme 22) [83,84,85]. Notably, when quinoline-containing heteroatropisomers **68** and **72** were treated with *m*-CPBA, the quinoline N-oxide could be generated with retained optical integrity [83,85]. In addition, the organocatalyzed aza-Michael/aldol cascade reaction between 2-aminoaryl ketones **73** and ynals **74** to access axially chiral quinoline-3-carbaldehydes **75** was also reported in 2021. Quinoline-carbaldehydes **75** were obtained in high yield with the assistance of diluted HCl(aq) which could promote aromatization in the last step (Scheme 23) [86,87]. Similarly, when 2-substituted phenols in 3-(naphthalene-1-yl) propionaldehyde were protected with a large protecting group such as 2,4,6-Me_3_-Bn, the axially chiral 2-arylquinolines **78** could be accessed by chiral amine (**L23**)-catalyzed heteroannulation between **77** and 2-aminobenzaldehyde **76** (Scheme 24) [88].

**Scheme 25:**
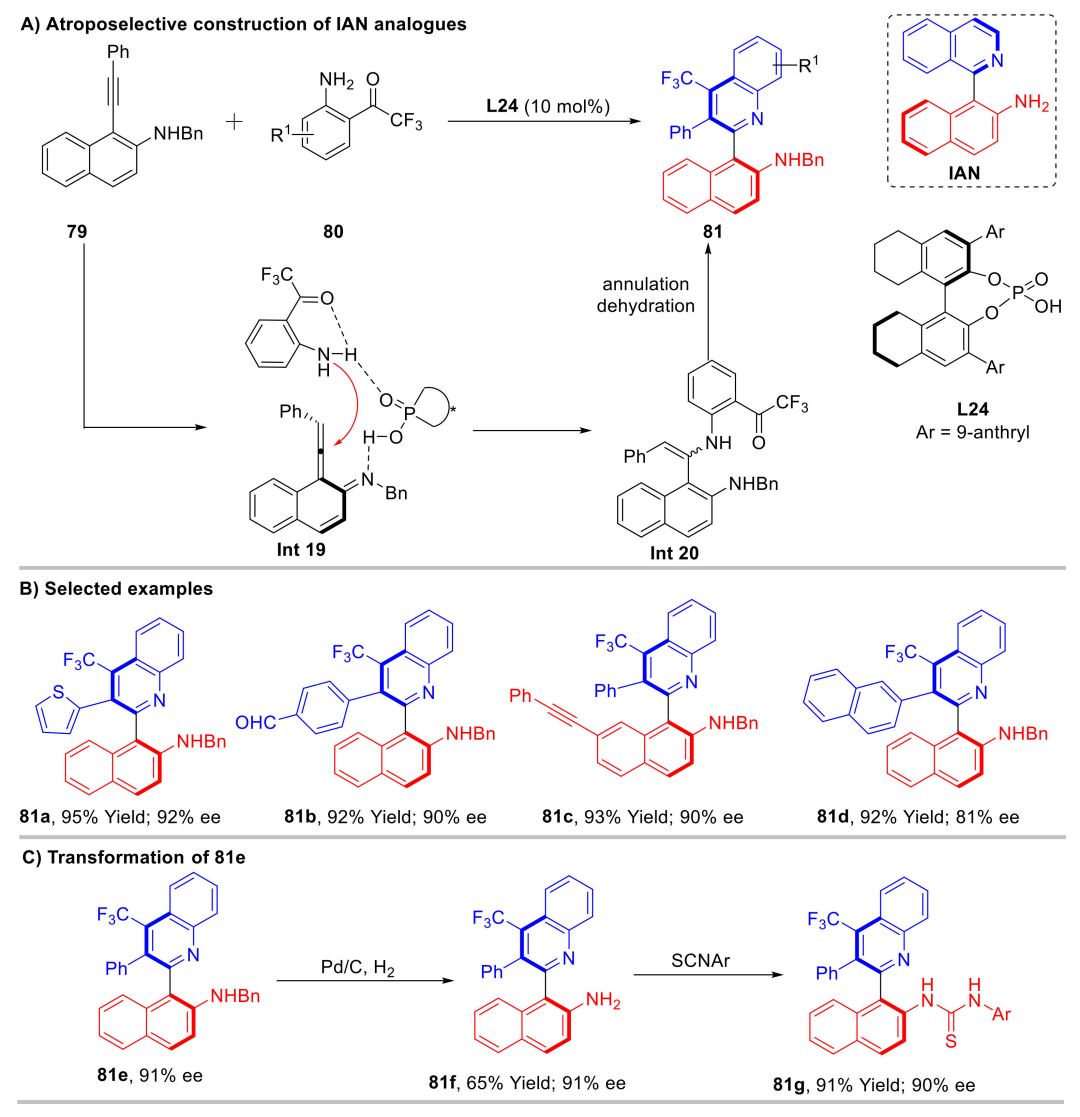
Atroposelective construction of IAN analogs.

In 2020, Tan and co-workers uncovered a highly efficient approach to providing the chair IAN (Isoquinoline and 2-Amino Naphthalene) analogs [89]. The reaction proceeds through organocatalytic asymmetric heteroannulation of ortho-alkynyl-naphthylamines **79**, to generate vinylidene orthoquinone methide (VQM) intermediates **Int 19**, which was then attacked by amine **80** to form **Int 20**. The intramolecular aldol condensation of **Int 20**, followed by dehydration delivered biologically important atropisomeric C2-arylquinoline skeleton **81** (Scheme 25) [90]. The benzyl (Bn) protecting group in **81e** was successfully removed by catalytic hydrogenation of **81f**, which could further react with isothiocyanate to produce the fascinating atropisomeric thiourea **81g**.

**Scheme 26:**
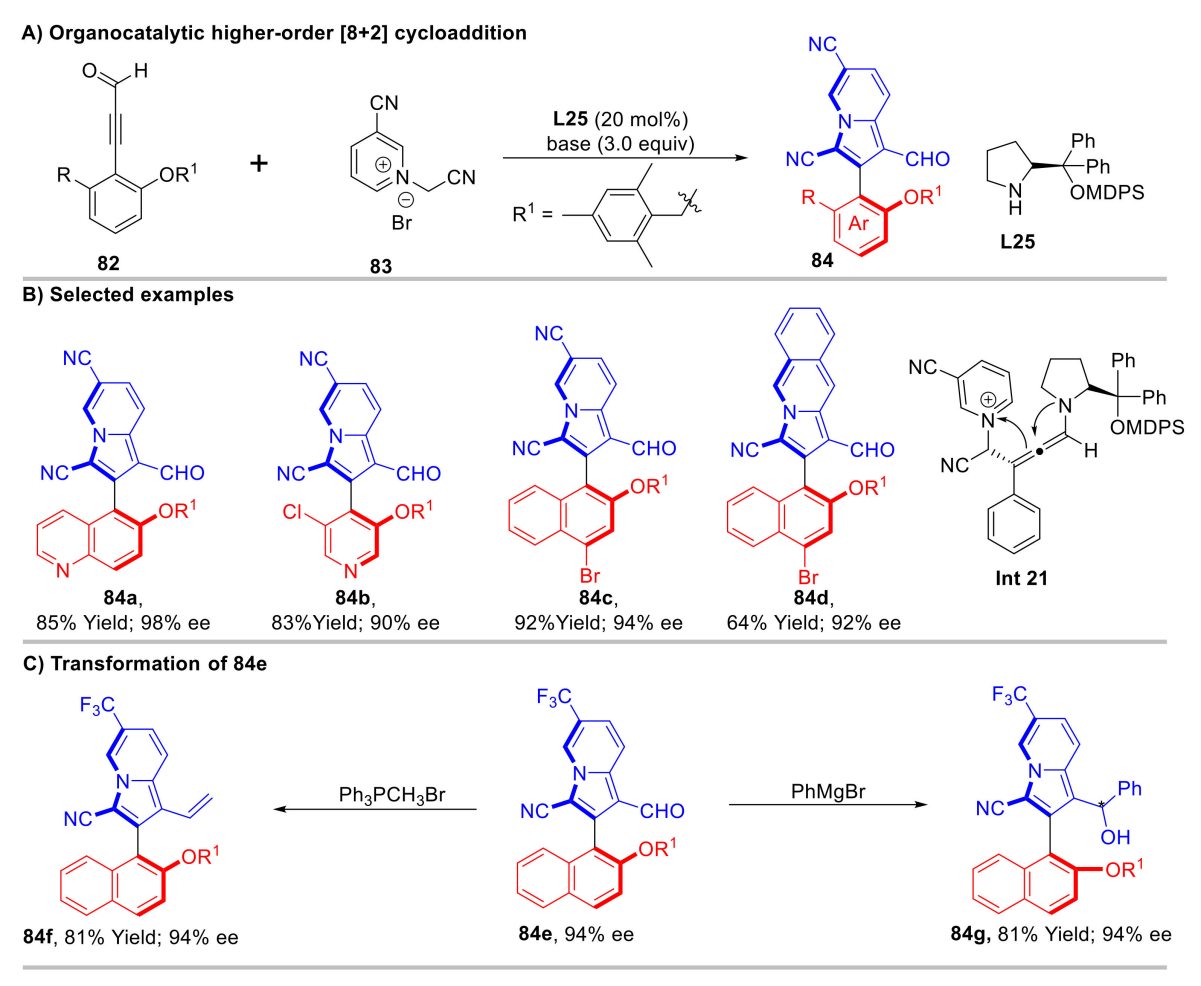
Organocatalytic higher-order [8 + 2] cycloaddition.

Later, Wang and co-workers presented the amine **L25**-catalyzed higher-order [8 + 2] cycloaddition of pyridinium ylides **83** with **82** via Michael addition and intramolecular cyclization, furnishing an array of 3-arylindolizines **84** with broad functional group compatibilities (Scheme 26) [91].

**Scheme 27:**
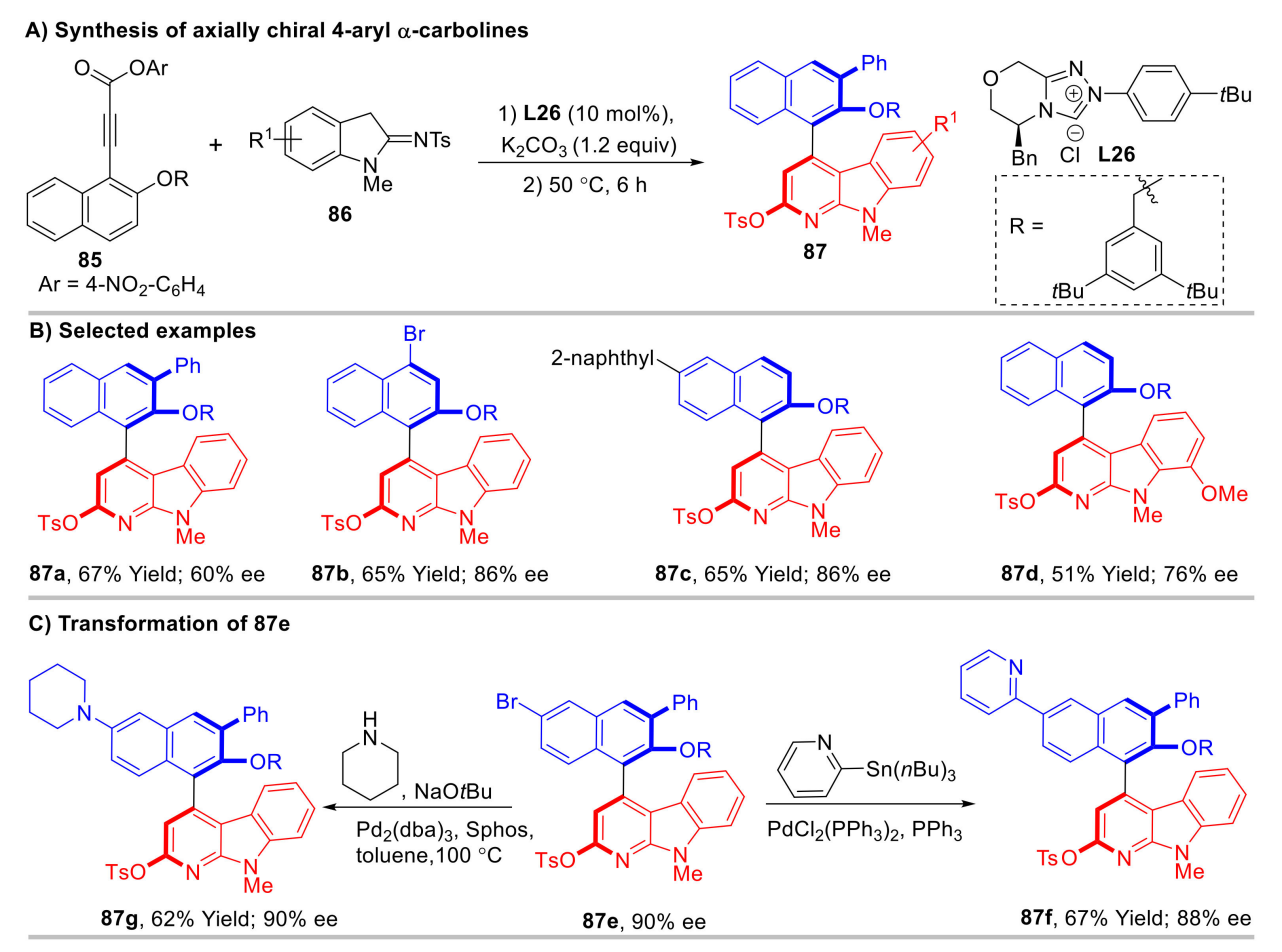
Synthesis of axially chiral 4-aryl α-carbolines.

Simultaneously, Du’s group successfully prepared axially chiral 4-aryl α-carbolines **87** through N-heterocyclic carbene (**L26**) catalyzed formal [3 + 3] annulation of 2-sulfonamidoindolines **86** with 4-nitrophenyl 3-arylpropiolates **85** (Scheme 27) [92]. The late-stage functionalization of the product was also demonstrated by the coupling reactions between **87e** and 2-(tributylstannyl) pyridine and piperidine under mild conditions.

**Scheme 28:**
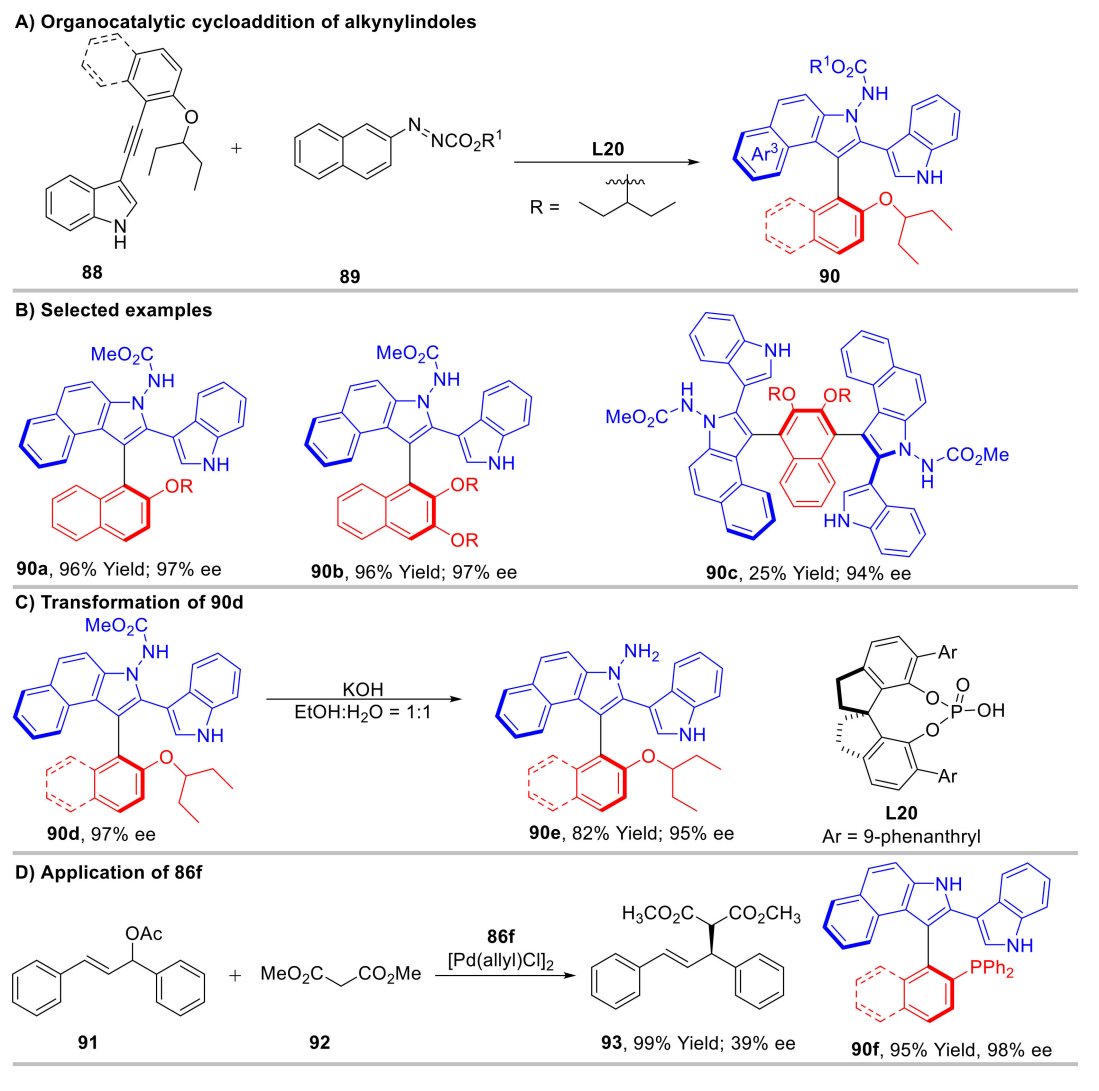
Organocatalytic cycloaddition of alkynylindoles.

Recently, the asymmetric cycloaddition of 3-alkynylindoles **88** with azonaphthalenes **89** catalyzed by 9-phenanthryl-substituted chiral phosphoric acid **L20** for the atroposelective preparation of new indole-based biaryl skeletons was developed by Zhou and co-workers (Scheme 28) [93]. A series of substituted indoles and naphthalenes could be used in the reaction for furnishing axially chiral arylindole derivatives **90** with excellent stereoselectivities. According to DFT calculations and control experiments, it was proposed that the new indole-based biaryl skeletons could be delivered via the dearomatization of an indole-triggered intramolecular aza-Michael addition cascade process. Moreover, the product **90e** could be converted into **90f** after a four-step transformation. Interestingly, **90f** could be engaged as a ligand to catalyze asymmetric allylation reaction between **91** and **92** to generate **93** with 99% yield and 39% ee.

**Scheme 29:**
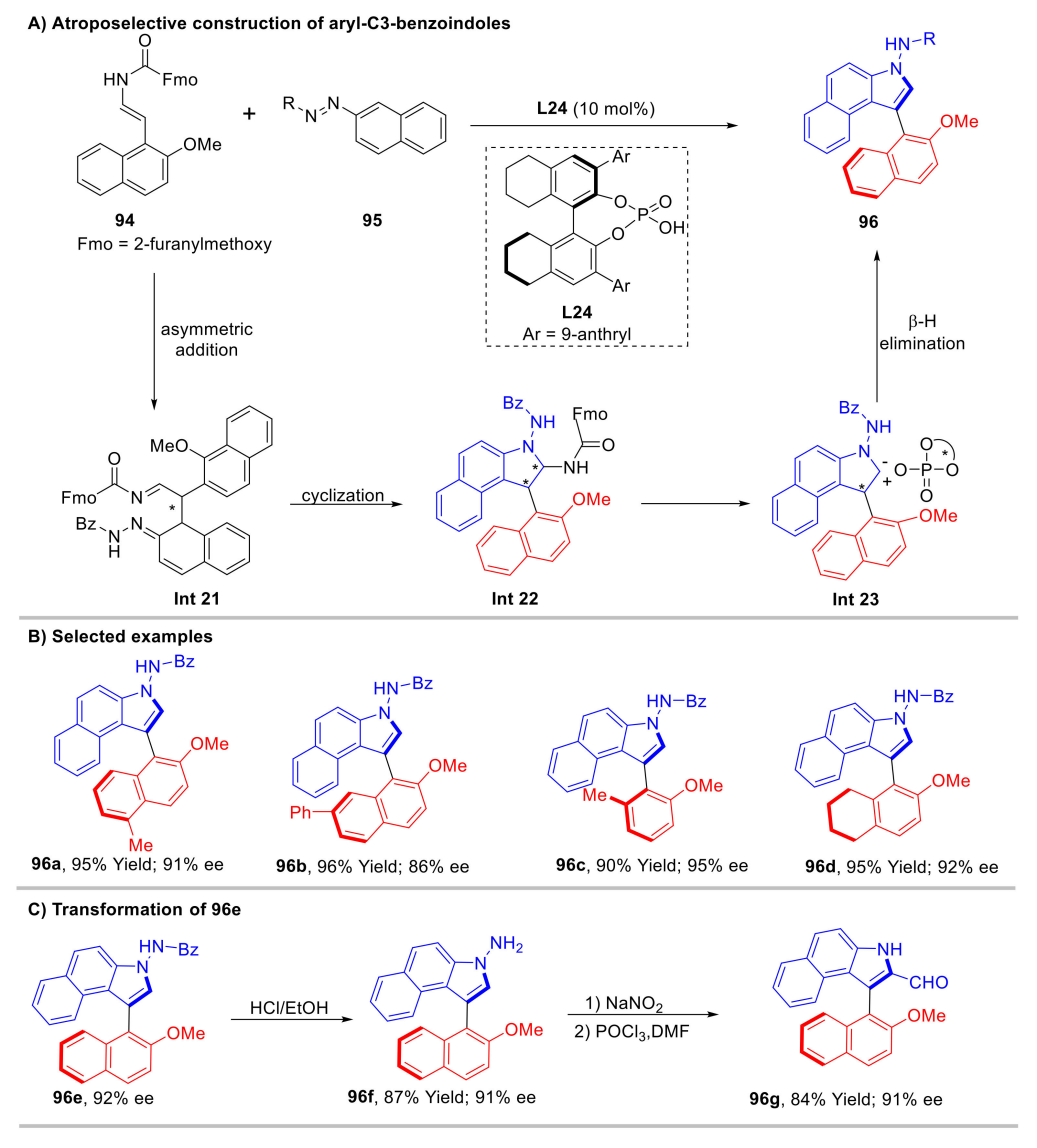
Atroposelective construction of aryl-C3-benzoindoles.

In addition to employing aryl alkynes in the construction of axially chiral biaryls, Zhou’s group also creatively used aryl ethylenes as the starting materials for the same goal. In this case, the asymmetric addition of phenol-derived enecarbamates **94** and azonaphthalenes **95** produced **Int 21**, which, upon intramolecular cyclization and elimination of a carbamate, generated **Int 23**. Lastly, β-H elimination of **Int 23** produced the axially chiral product **96** (Scheme 29) [94]. Further studies indicated that the catalyst **L24** plays a decisive role in controlling the enantioselectivity and the reactivity. Axially chiral product **96f** could be smoothly converted into **96g** in the presence of phosphorus oxychloride and DMF.

**Scheme 30:**
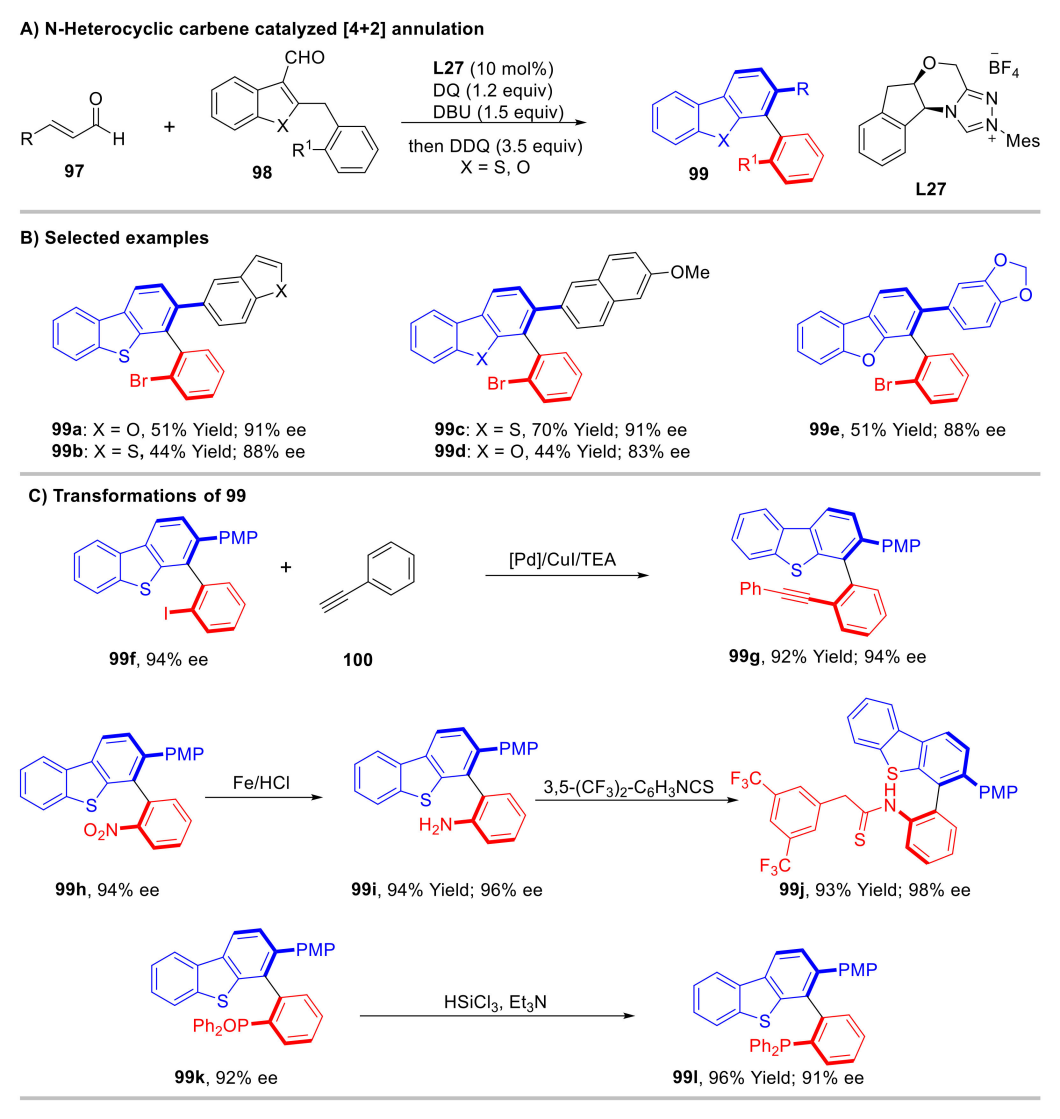
N-Heterocyclic carbene catalyzed [4 + 2] annulation.

In the same year, an *N*-heterocyclic carbene (**L27**) catalyzed [4 + 2] annulation of enals **97** with 2-benzylbenzothiophene **98** was developed for enantioselective de novo construction of ortho-substituted biaryl **99** (Scheme 30) [95]. A novel axially chiral benzothiophene-fused biaryl **99** could be obtained through central-to-axial chirality conversion via a decarbonylation-aromatization cascade process. Moreover, biaryl products **99f**, **99h**, and **99k** could be converted into synthetic useful biaryls **99g**, **99j**, and **99l** through a simple transformation in one or two steps.

**Scheme 31:**
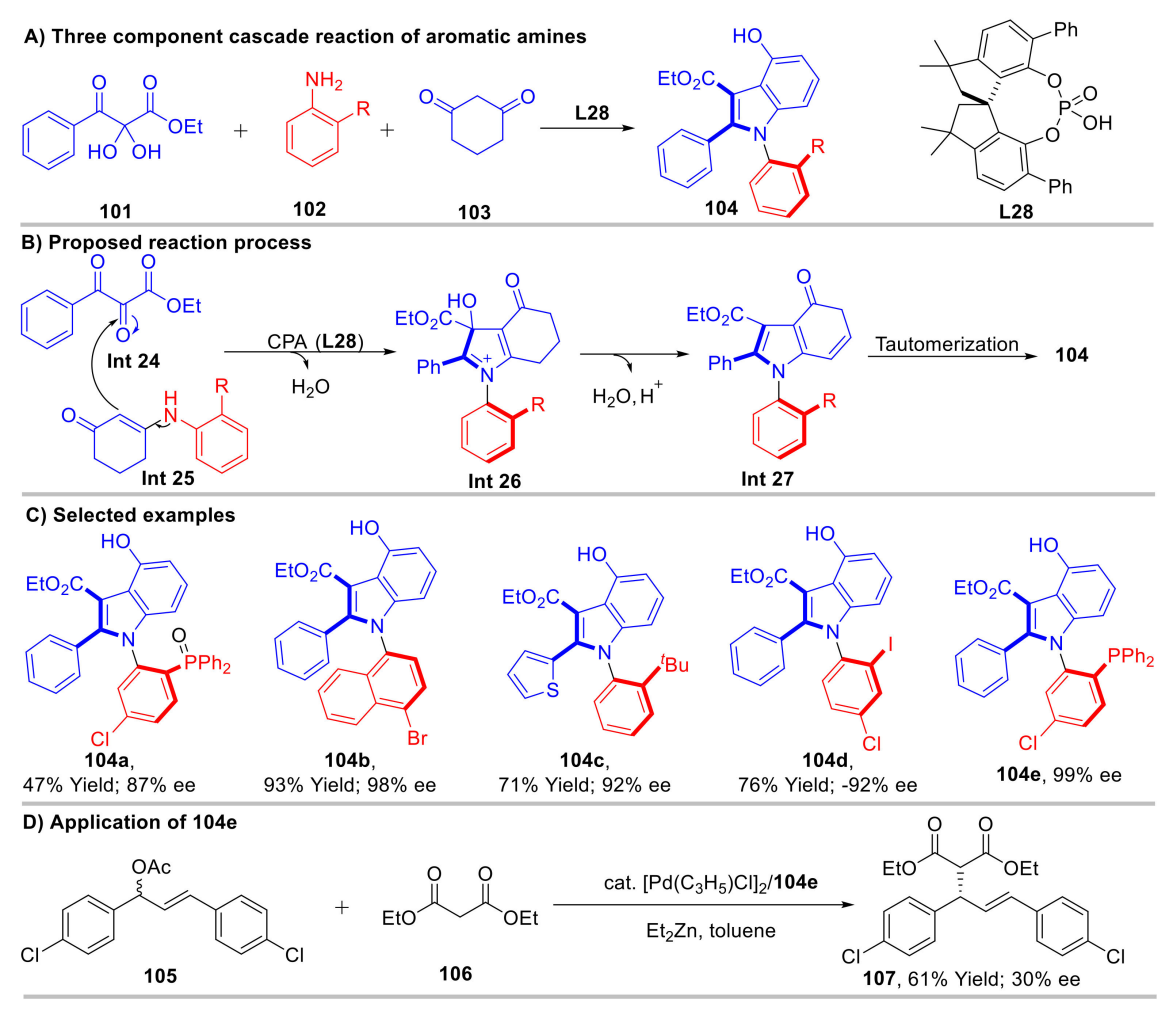
Three-component cascade reaction of aromatic amines.

**Scheme 32:**
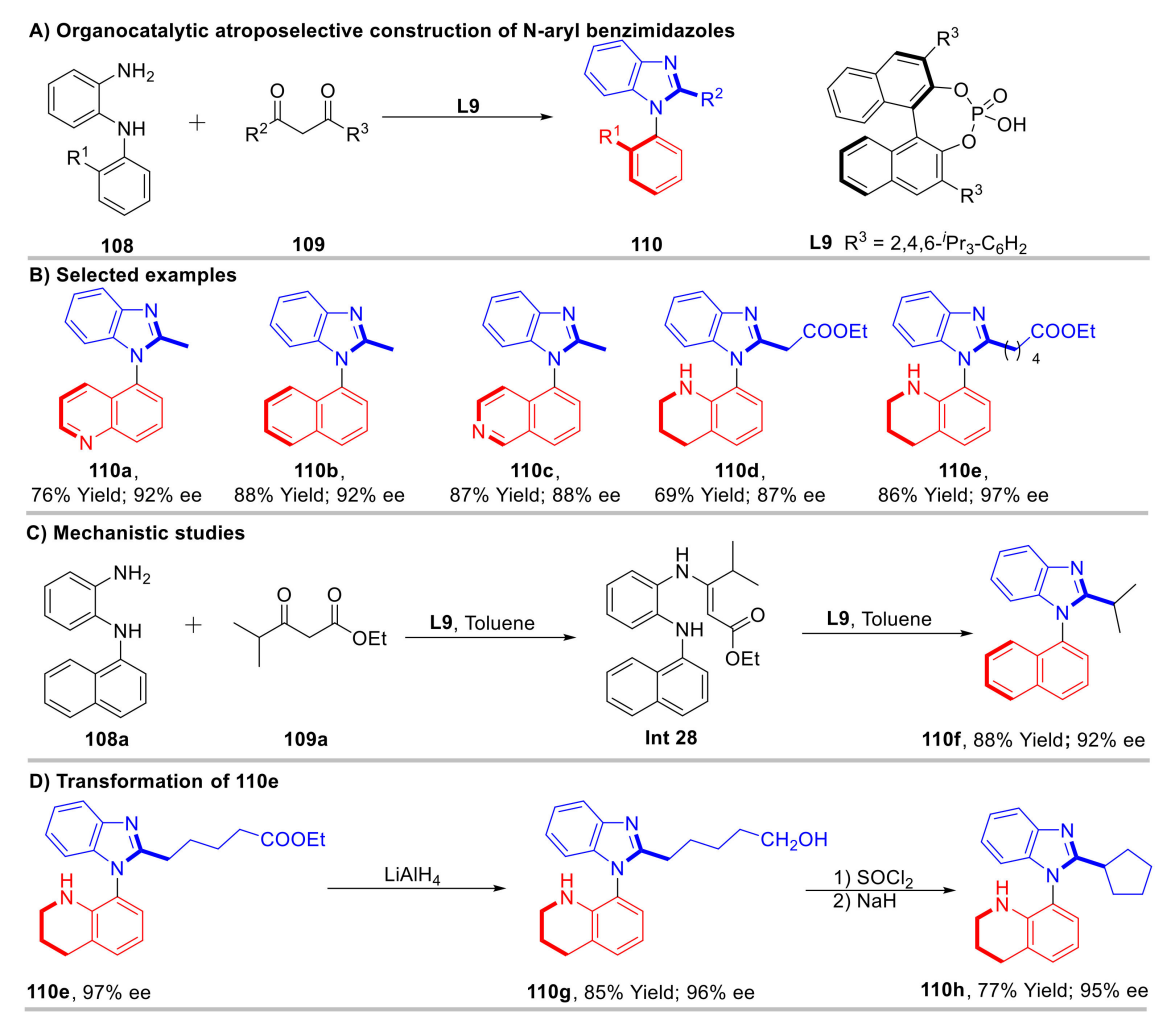
Organocatalytic atroposelective construction of *N*-aryl benzimidazoles.

Furthermore, a chiral spirocyclic phosphoric acid (**L28**)-catalyzed three-component cascade reaction of 2,3-diketoesters (**101**), aromatic amines (**102**), and 1,3-cyclohexanediones (**103**) has also been applied to construct the axially chiral N-arylindoles **104** (Scheme 31) [96]. This process went through the initial aldol condensation between enamine intermediate **Int 25** with **Int 24** mediated by **L28**, which was followed by dehydrative cyclization, 1,4-elimination, and tautomerization to generate the desired product **104**. In addition, product **104e** could be used as a novel ligand in a Pd-catalyzed asymmetric Tsuji–Trost allylation reaction. In a related report, Fu’s group demonstrated that axially chiral N-aryl benzimidazoles scaffolds could be produced via the reaction of 1,3-dicarbonyl compounds **109** with 1,2-diamines **108** via carbon-carbon bond cleavage catalyzed by CPA (chiral phosphoric acid) **L9** (Scheme 32) [97]. The enantioselectivity of **110** could be improved by using molecular sieve as the additive. In addition, 1,2,3,4-tetrahydroquinolin-8-yl, isoquinoline-5-yl, and quinoline-5-yl benzimidazole derivatives could be achieved under this condition with high yield (up to 86%) and excellent enantioselectivity (up to 98% ee). Further studies indicated that enamine **Int 28** was the key intermediate in this transformation. In addition, the reduction of ester **110e** with LiAlH_4_ could deliver alcohol **110g**, which, upon treatment with SOCl_2_, followed by NaH triggered intramolecular cyclization, producing cyclic product **110h**.

### 3.2. Intramolecular Cyclization Reaction Catalyzed by Organo-Catalysis

#### 3.2.1. Enantioselective Synthesis of Heteroaromatic Chiral Skeleton with a Five-Membered Ring

**Scheme 33:**
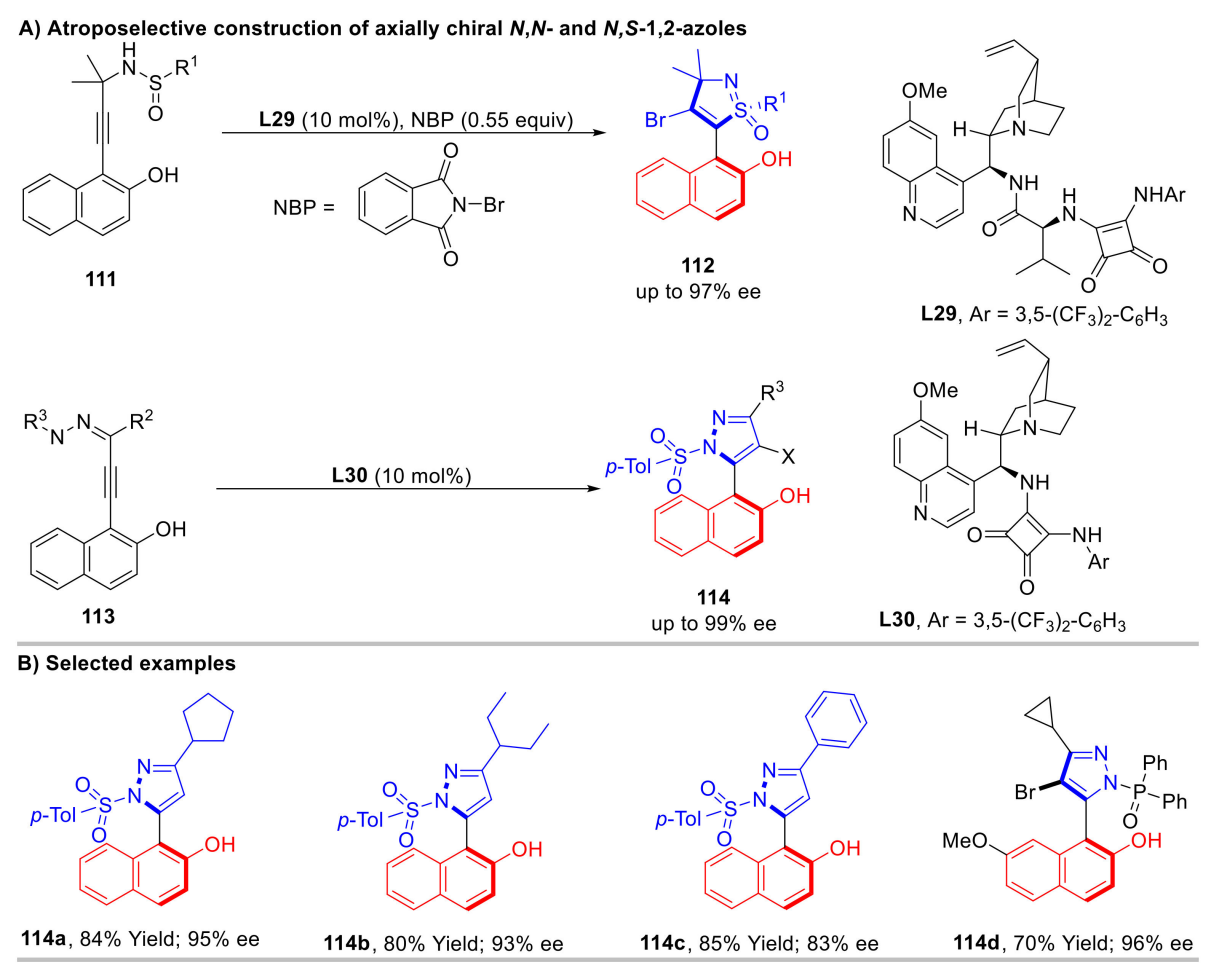
Atroposelective construction of axially chiral *N*,*N*- and *N*,*S*-1,2-azoles.

Diverse atropisomeric heterobiaryl molecules including axially chiral *N*,*N*- and *N*,*S*- 1,2-azole scaffolds were synthesized utilizing modified vinylidene orth*o*-quinone methide precursors **111** and **113** via a ring formation method catalyzed by cinchona alkaloids-derived squalamine **L29** and **L30** (Scheme 33) [98]. This work, developed by Yan’s group, simultaneously controlled the atroposelective installation of a stereogenic axis and the formation of a heterocyclic ring. The corresponding axially chiral naphthyl-isothiazole S-oxides **112** were synthesized by this methodology via a kinetic resolution process with high *S* factors in the presence of *N*-bromophthalimide (NBP). Meanwhile, when hydrazone **113** instead of **111** was employed as the starting material, in the presence of a slightly different catalyst (**L30**), naphthyl-pyrazoles **114** could be furnished in the absence of NBP.

#### 3.2.2. Enantioselective Synthesis of Heteroaromatic Chiral Skeleton with a Fused Ring

**Scheme 34:**
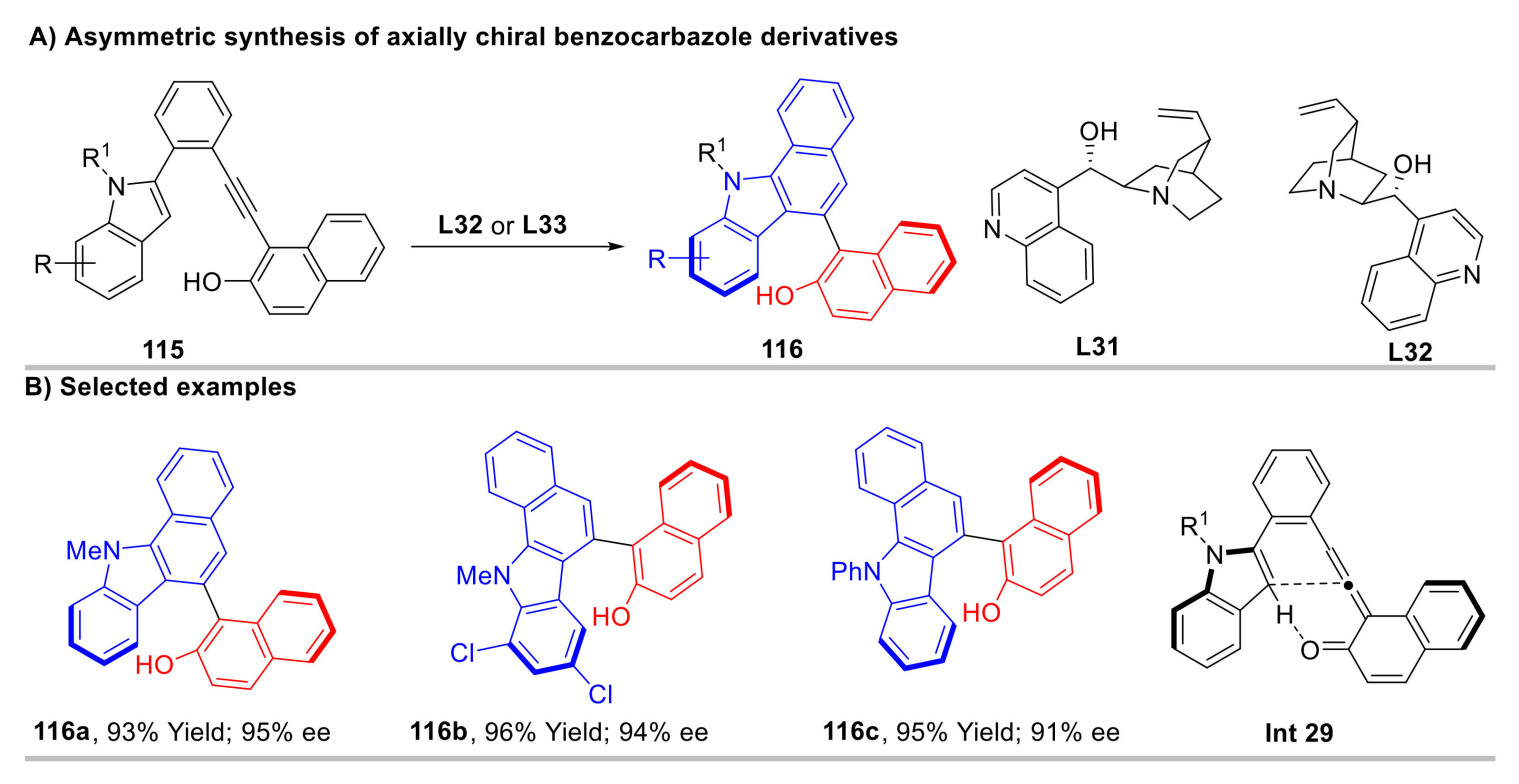
Asymmetric synthesis of axially chiral benzocarbazole derivatives.

In recent years, vinylidene ortho-quinone methides precursors were engaged as the powerful building blocks to construct different kinds of atropisomeric heterocycles. In 2018, Irie’s group described an unprecedented approach to access novel benzocarbazole derivatives **116** through enantioselective hydroarylation of alkynes **115** under transition-metal-free conditions (Scheme 34) [99]. It was found that chiral vinylidene o-quinone methide (VQM) intermediate **Int 29** could be generated by a proton shift of **115** catalyzed by cinchonine **L31** or cinchonidine **L32**.

**Scheme 35:**
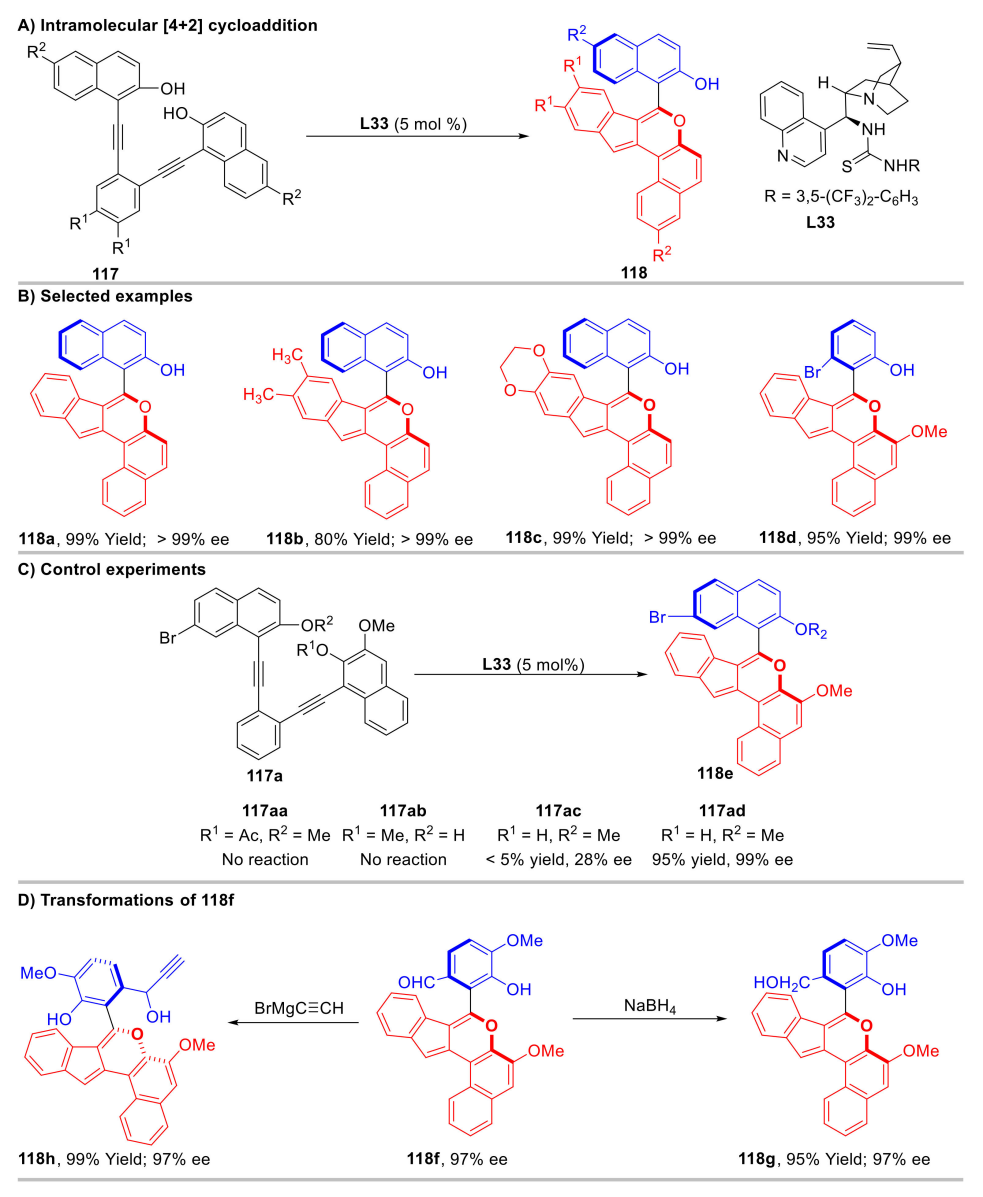
Intramolecular [4 + 2] cycloaddition.

In the same year, 2-ethynylphenol derivatives **117** were employed as the substrates to furnish furan atropisomers **118** with various functional groups catalyzed by quinine-derived thiourea **L33**, through an atroposelective intramolecular [4 + 2] cycloaddition via ortho-quinone methide intermediates (Scheme 35) [100]. Control experiments indicated that the hydroxyl group played a key role in increasing yield and controlling the enantioselectivity in this transformation, as lacking either the right-hand phenolic hydroxyl group or both of the two free OH groups led to no reaction taking place. Further functional group transformations were realized by the reactions of **118f** with NaBH_4_ or Grignard reagent to afford **118g** and **118h**.

**Scheme 36:**
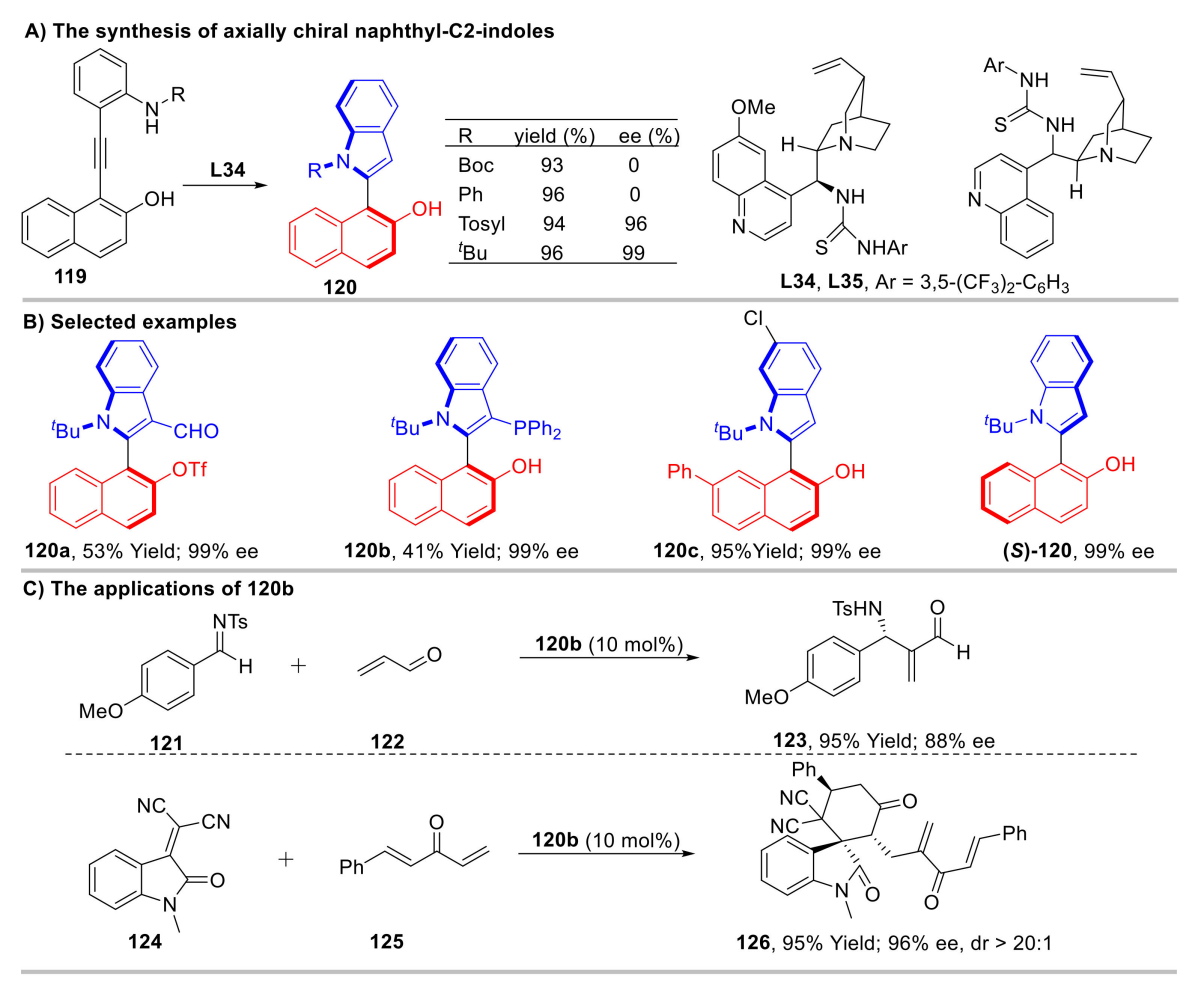
The synthesis of axially chiral naphthyl-C2-indoles.

In 2019, Yan and co-workers developed a chiral Brønsted base quinine thiourea **L34**, catalyzed cycloaddition of ortho-alkynylanilines **119** for the synthesis of naphthyl-C2-indoles **120** (Scheme 36) [101]. Interestingly, when the cinchonine-derived thiourea catalyst **L35** which had an identical stereoconfiguration with **L34** was used as the catalyst, the opposite enantioselectivity could be achieved with 99% ee. It is worth noting that aniline bearing different protecting groups has a great influence on the enantioselectivity, even though the yields of the transformation were not affected. Furthermore, by this methodology, the indole derivatives such as **120a** with CHO and **120b** with chiral phosphine could be obtained with excellent ee values. In addition, **120b** could be used as an organocatalyst to catalyze asymmetric aza-Baylis–Hillman reaction and [4 + 2] cyclization reaction affording the corresponding products **123** and **126** in good yield with high enantioselectivity.

**Scheme 37:**
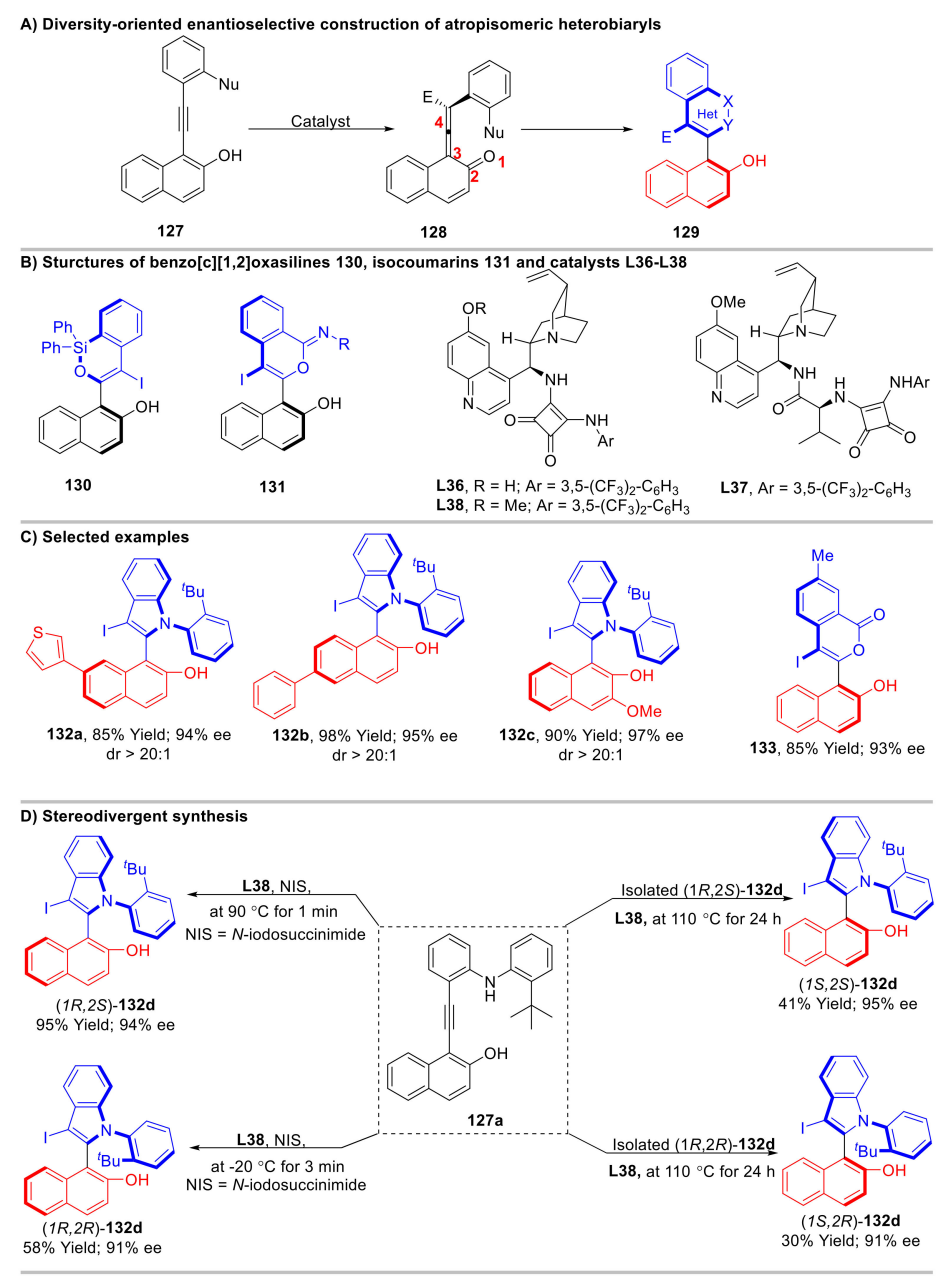
Diversity-oriented enantioselective construction of atropisomeric heterobiaryls.

Very recently, the same group disclosed a methodology for the construction of atropisomeric heterobiaryl structures including benzo[*c*][1,2]oxasilines **130** and isocoumarins **131** from simple starting materials **127** via asymmetric intramolecular annulation mediated by catalyst **L36** and **L37** (Scheme 37) [102]. Significantly, four isomers {(*1R*, *1S*)-**132d**; (*1R*, *1R*)-**132d**; (*1S*, *1S*)-**132d;** (*1S*, *1R*)-**132d**} of the annulation products from **127a** could be achieved by using **L38** as the catalyst through control of the reaction time and temperature. Control experiments showed that when the hydroxyl group of **127** was protected, axially chiral heterocyclic architecture could not be produced because the formation of the VQM intermediate **128** was inhibited.

**Scheme 38:**
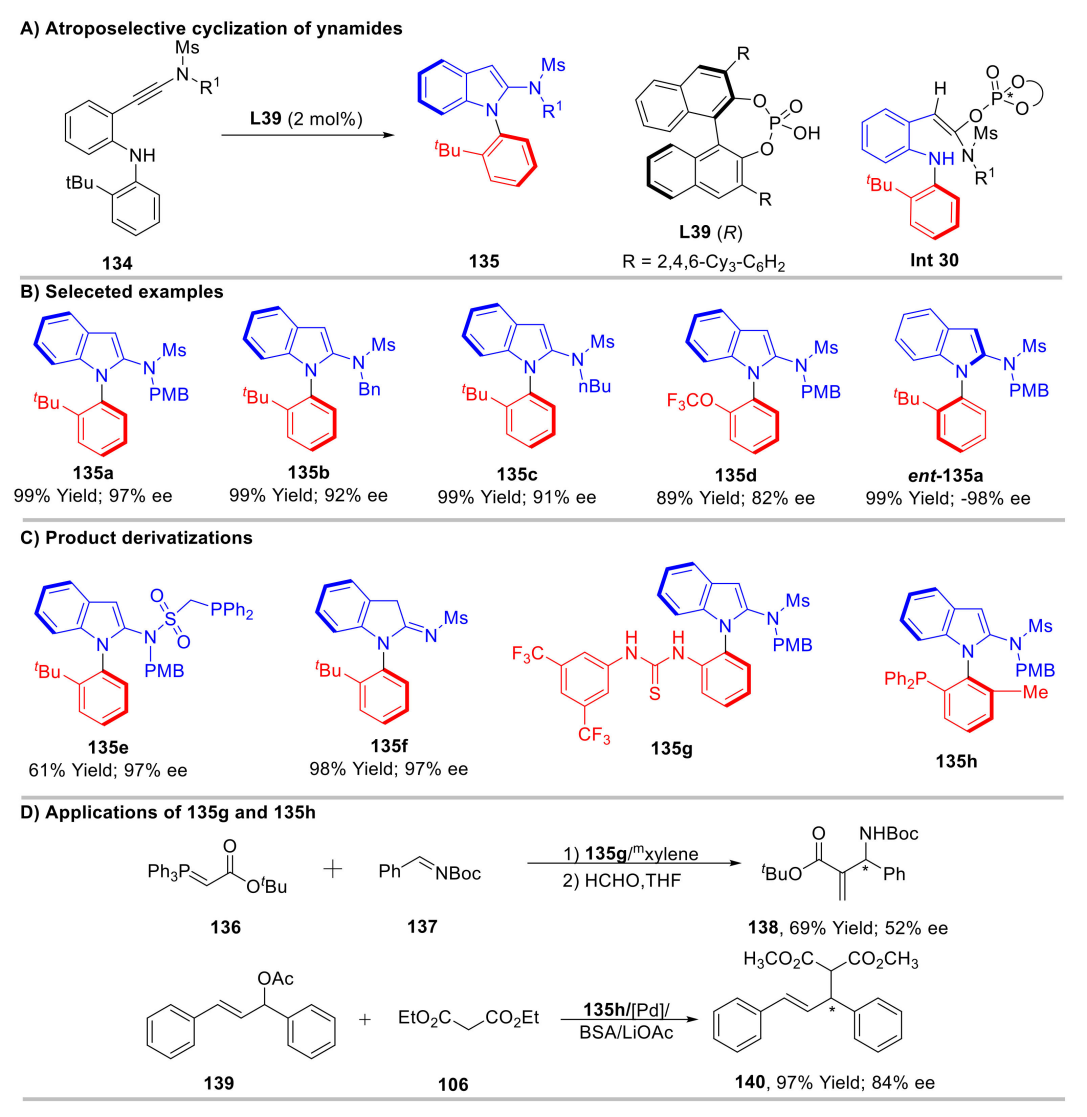
Atroposelective cyclization of ynamides.

Recently, the first example of 5-endo-dig annulation of *o*-anilino ynamides **134** was developed by Ye and co-workers to generate axially chiral *N*-arylindole framework **135** via **Int 30** catalyzed by chiral phosphoric acid (*R*)-**L39** (Scheme 38) [103]. Notably, when (*S*)-**L39** was employed, the **ent-135a** could be obtained with an excellent ee value. The obtained products could be transformed into a range of other analogs in one to two steps. Furthermore, the corresponding thiourea **135g** and phosphine **135h** furnished by a two-step transformation, could serve as an organocatalyst or ligand for other applications, such as asymmetric addition reaction between **136** and N-Boc imine **137**, and Pd-catalyzed allylic alkylation of **139** with **106**, to produce **138** and **140** respectively.

**Scheme 39:**
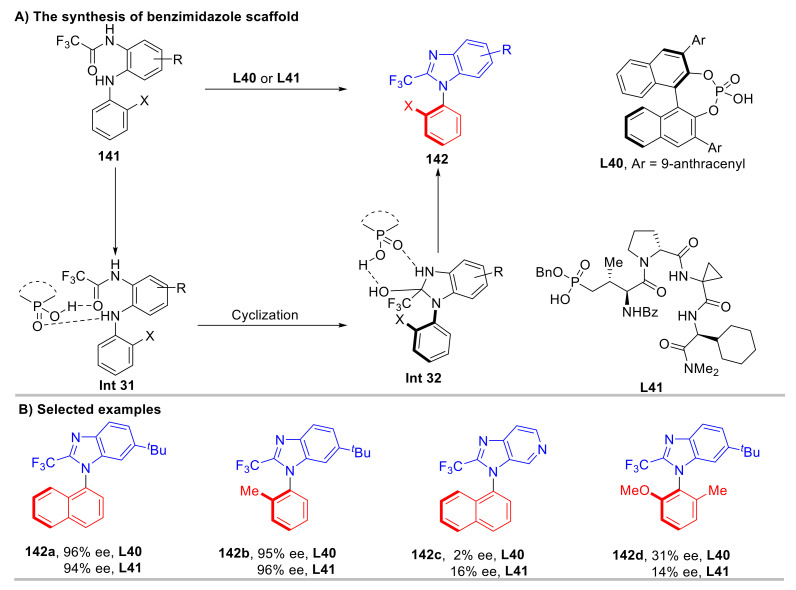
The synthesis of axially chiral benzimidazole scaffold.

Miller’s group disclosed an atroposelective cyclodehydration process of 1,1,1-trifluoro-3-(2-(phenylamino) phenyl) propane-2-one **141** with the assistance of C2-symmetric type catalyst **L40** or peptide-based catalyst **L41** (Scheme 39) [104]. The products with o,o′-disubstituted or 3H-imidazo [4,5-c]pyridines were obtained in moderate to excellent enantioselectivity. DFT calculations showed that the global conformation of the peptide **L41** dominated the enantioselectivity of this transformation.

The above-mentioned studies clearly demonstrated that chiral phosphoric acid, chiral amine, chiral cinchona alkaloids, and N-heterocyclic carbene are highly efficient catalysts to promote the formation of heterobiaryl skeletons. Arylquinoline and heterocycles bearing O, S, Si, B, and N could be furnished by using 2-aminoaryl ketone and vinylidene orthoquinone methide precursor as the reaction substrate. Moreover, axially chiral helical compounds and heterobiaryls could be obtained with excellent yield and ee values.

## 4. Conclusions and Perspective

In recent years, the atroposelective synthesis of biaryls has undergone significant progress with the rapid development of asymmetric reactions, which in turn realized a range of new methodologies for synthesizing various heterobiaryl scaffolds with C-N, C-C, and N-N chiral axes mediated by transition-metal and (or) organic catalysts. De novo synthesis of these motifs has thus become a representative and powerful strategy to provide axially chiral heterobiaryl skeletons containing indoles, pyrroles, triazoles, etc. Translation of de novo synthesis mediated by transition-metal into atroposelective variants has been established forming the indole-based framework and heterobiaryl atropisomers containing the C-N chiral axis. Meanwhile, the breakthrough in the use of organocatalysts in the de novo synthesis strategy enabled the direct forging of stereogenic aryl-(hetero)aryl bonds furnishing axially chiral helical compounds and atropisomeric quinoline scaffold through stereocontrolled annulation. Furthermore, the de novo synthesis strategy offers a new idea for the synthesis of heterobiaryls bearing an *N*-*N* chiral axis. The highly convergent and superior atom economy, in addition to a wide range of different reaction patterns, further broaden structural diversity in the products. A series of novel optically pure molecules were obtained based on these protocols, which offer opportunities for further asymmetric transformations and the discovery of new drug molecules. Nevertheless, there remain many challenges in this field. For example, compared to heterobiaryl molecules with C-N and C-C chiral axes, axially chiral compounds with an N-N chiral axis are much less developed because of the lower rotating energy barrier, and thus, are rarely reported. Also, presently, most reactions used to synthesize axially chiral biaryls skeletons are carried out at larger catalyst loadings (2 mol% to 20 mol%) with relatively complex starting materials, which hinders their application in industry and drug discovery. Therefore, new annulation reactions with readily accessible substrates under small catalyst loading remain highly desirable. Furthermore, the development of modern organic chemistry needs greener and more sustainable processes such as photocatalytic, electrocatalytic, and biocatalytic synthesis; however, these strategies are barely applied in the construction of axially chiral heterobiaryl skeletons. In addition, more attention should be paid to the application of heterobiaryl skeletons in asymmetric synthesis and drug development to further facilitate the discovery of new, greener, and highly efficient protocols for this topic.

## Data Availability

Not applicable.

## Sample Availability

Samples of the compounds are not available from the authors.

## References

[1] Wipf P., Skoda E.M., Mann A., Wermuth C.G., Aldous D., Raboisson P., Rognan D. (2015). Conformational Restriction and Steric Hindrance in Medicinal Chemistry. The Practice of Medicinal Chemistry.

[2] Kuhn R., Freudenberg K. (1933). Molekulare Asymmetrie. Stereochemie.

[3] Oki M. (1983). Recent Advances in Atropisomerism. Top. Stereochem..

[4] Christie G.H., Kenner J. (1922). The Molecular Configurations of Polynuclear Aromatic Compounds. J. Chem. Soc. Trans..

[5] Miyashita A., Yasuda A., Takaya H., Toriumi K., Ito T., Souchi T., Noyori R. (1980). Synthesis of 2,2′-Bis(diphenylphosphino)-1,1′-binaphthyl (BINAP), an Atropisomeric Chiral Bis(triaryl)phosphine, and Its Use in the Rhodium(I)-Catalyzed Asymmetric Hydrogenation of.alpha.-(Acylamino)acrylic Acids. J. Am. Chem. Soc..

[6] Xiao X., Chen B., Yao Y.P., Zhou H.J., Wang X., Wang N.Z., Chen F.E. (2022). Construction of Non-Biaryl Atropisomeric Amide Scaffolds Bearing a C–N Axis via Enantioselective Catalysis. Molecules..

[7] Wang Y.-B., Tan B. (2018). Construction of Axially Chiral Compounds via Asymmetric Organocatalysis. Acc. Chem. Res..

[8] Qin W.L., Liu Y.D., Yan H.L. (2022). Enantioselective Synthesis of Atropisomers via Vinylidene orthoQuinone Methides (VQMs). Acc. Chem. Res..

[9] Shao Y.D., Cheng D.J. (2021). Chiral Phosphoric Acid: A Powerful Organocatalyst for the Asymmetric Synthesis of Heterocycles with Chiral Atropisomerism. ChemCatChem.

[10] Cheng J.K., Xiang S.-H., Li S., Ye L., Tan B. (2021). Recent Advances in Catalytic Asymmetric Construction of Atropisomers. Chem. Rev..

[11] Carmona J.A., Rodríguez-Franco C., Fernández R., Hornillos V., Lassaletta J.M. (2021). Atroposelective Transformation of Axially Chiral (hetero) Biaryls. From Desymmetrization to Modern Resolution Strategies. Chem. Soc. Rev..

[12] Cheng D.-J., Shao Y.-D. (2020). Advances in the Catalytic Asymmetric Synthesis of Atropisomeric Hexatomic N-Heterobiaryls. Adv. Synth. Catal..

[13] Tajuddeen N., Feineis D., Ihmels H., Bringmann G. (2022). The Stereoselective Total Synthesis of Axially Chiral Naphthylisoquinoline Alkaloids. Acc. Chem. Res..

[14] Norton R.S., Wells R.J. (1982). A Series of Chiral Polybrominated Biindoles from the Marine Blue-Green Alga Rivularia Firma. Application of Carbon-13 NMR Spin-Lattice Relaxation Data and Carbon-13-Proton Coupling Constants to Structure Elucidation. J. Am. Chem. Soc..

[15] Ito C., Thoyama Y., Omura M., Kjiura I., Furukawa H. (1993). Alkaloidal Constituents of Murraya koenigii. Isolation and Structural Elucidation of Novel Binary Carbazolequinones and Carbazole Alkaloids. Chem. Pharm. Bull..

[16] Hughes C.C., Pietro-Davo A., Jensen P.R., Fenical W. (2008). The Marinopyrroles, Antibiotics of an Unprecedented Structure Class from a Marine Streptomyces sp.. Org. Lett..

[17] Toenjes S.T., Gustafson J.L. (2018). Atropisomerism in Medicinal Chemistry: Challenges and Opportunities. Future Med. Chem..

[18] Basilaia M., Chen M.H., Secka J., Gustafson J.L. (2022). Atropisomerism in the Pharmaceutically Relevant Realm. Acc. Chem. Res..

[19] Wang J., Zeng W., Li S., Shen L., Gu Z., Zhang Y., Li J., Chen S., Jia X. (2017). Discovery and Assessment of Atropisomers of (±)-Lesinurad. ACS Med. Chem. Lett..

[20] Lanman B.A., Allen J.R., Allen J.G., Amegadzie A.K., Ashton K.S., Booker S.K., Chen J.J., Chen N., Frohn M.J., Goodman G. (2020). Discovery of a Covalent Inhibitor of KRASG12C (AMG 510) for the Treatment of Solid Tumors. J. Med. Chem..

[21] Brown D.G., Boström J. (2016). Analysis of Past and Present Synthetic Methodologies on Medicinal Chemistry: Where Have All the New Reactions Gone?. J. Med. Chem..

[22] Rokade B.V., Guiry P.V. (2018). Axially Chiral P, N-Ligands: Some Recent Twists and Turns. ACS Catal..

[23] Malkov A.V., Dufková L., Farrugia L., Kočovský P. (2003). Quinox, a Quinoline-Type N-Oxide, as Organocatalyst in the Asymmetric Allylation of Aromatic Aldehydes with Allyltrichlorosilanes: The Role of Arene–Arene Interactions. Angew. Chem. Int. Ed..

[24] Ramírez-López P., Ros A., Estepa B., Fernandez R., Lassaletta J.M. (2016). A Dynamic Kinetic C–P Cross–Coupling for the Asymmetric Synthesis of Axially Chiral P, N Ligands. ACS Catal..

[25] Biswas A., Pan S., Samanta R. (2022). Cu(II)-Catalyzed Construction of Heterobiaryls Using 1-Diazonaphthoquinones: A General Strategy for the Synthesis of QUINOX and Related *P*,*N* Ligands. Org. Lett..

[26] Jiang P.-Y., Fan K.-F., Li S., Xiang S.-H., Tan B. (2021). Metal-Free Oxidative Cross-Coupling Enabled Practical Synthesis of Atropisomeric QUINOL and its Derivatives. Nat. Commun..

[27] Katona D., Lu Y.P., Li Y.X., Pullarkat S.A., Leung P.H. (2020). Catalytic Approach toward Chiral P, N-Chelate Complexes Utilizing the Asymmetric Hydrophosphination Protocol. Inorg. Chem..

[28] Bonne D., Rodriguez J. (2018). A Bird’s Eye View of Atropisomers Featuring a Five-Membered Ring. Eur. J. Org. Chem..

[29] Li Z.T., Liu S.J., Tan W., Shi F. (2020). Catalytic Asymmetric Construction of Axially Chiral Indole-Based Frameworks: An Emerging Area. Chem. Eur. J..

[30] He X.L., Wang C., Wen Y.W., Wang Z.Y., Qian S. (2021). Recent Advances in Catalytic Atroposelective Construction of Pentatomic Heterobiaryl Scaffolds. ChemCatChem.

[31] Shaaban S., Li H.H., Otte F., Strohmann C., Antonchick A.P., Waldmann H. (2020). Enantioselective Synthesis of Five-Membered-Ring Atropisomers with a Chiral Rh(III) Complex. Org. Lett..

[32] Luo J., Zhang T., Wang L., Liao G., Yao Q.J., Wu Y.J., Zhan B.B., Lan Y., Lin X.F., Shi B.F. (2019). Enantioselective Synthesis of Biaryl Atropisomers by Pd-Catalyzed C−H Olefination Using Chiral Spiro Phosphoric Acid Ligands. Angew. Chem. Int. Ed..

[33] Nguyen Q.-H., Guo S.-M., Royal T., Baudoin O., Cramer N. (2020). Intermolecular Palladium(0)-Catalyzed Atropo-enantioselective C-H Arylation of Heteroarenes. J. Am. Chem. Soc..

[34] Pan C.-Q., Yin S.-Y., Wang S.-B., Gu Q., You S.-L. (2021). Oxygen linked Cyclopentadienyl Rh(III) Complexes Catalyzed Asymmetric C−H Arylation of Benzo[h]quinolines with 1-Diazonaphthoquinones. Angew. Chem. Int. Ed..

[35] Woźniak Ł., Cramer N. (2021). Atropo-Enantioselective Oxidation Enabled Iridium(III)-Catalyzed C−H Arylations with Aryl Boronic Esters. Angew. Chem. Int. Ed..

[36] Yamaguchi K., Kondo H., Yamaguchi J., Itami K. (2013). Aromatic C–H Coupling with Hindered Arylboronic Acids by Pd/Fe Dual Catalysts. Chem. Sci..

[37] Zhang S., Yao Q.J., Liao G., Li X., Li H., Chen H.M., Hong X., Shi B.F. (2019). Enantioselective Synthesis of Atropisomers Fea-turing Pentatomic Heteroaromatics by Pd-Catalyzed C–H Alkynylation. ACS Catal..

[38] Dherbassy Q., Djukic J.-P., Wencel-Delord J., Colobert F. (2018). Two Stereoinduction Events in One C−H Activation Step: A Route towards Terphenyl Ligands with Two Atropisomeric Axes. Angew. Chem. Int. Ed..

[39] Zhan B.-B., Wang L., Luo J., Lin X.-F., Shi B.-F. (2020). Synthesis of Axially Chiral Biaryl-2-amines by PdII Catalyzed FreeAmine-Directed Atroposelective C−H Olefination. Angew. Chem. Int. Ed..

[40] Ma G.Y., Sibi M.P. (2015). Catalytic Kinetic Resolution of Biaryl Compounds. Chem. Eur. J..

[41] Wang J., Qi X., Min X.-L., Yi W., Liu P., He Y. (2021). Tandem Iridium Catalysis as a General Strategy for Atroposelective Construction of Axially Chiral Styrenes. J. Am. Chem. Soc..

[42] Koshino S., Takikawa A., Ishida K., Tanigushi T., Monde K., Kwon E., Umemiya S., Hayashi Y. (2020). Inversion of the Axial Information during Oxidative Aromatization in the Synthesis of Axially Chiral Biaryls with Organocatalysis as a Key Step. Chem. Eur. J..

[43] Qiu S., Gao X., Zhu S. (2021). Dirhodium(II)-Catalysed Cycloisomerization of Azaenyne: Rapid Assembly of Centrally and Axially Chiral Isoindazole Frameworks. Chem. Sci..

[44] Li H., Yan X., Zhang J., Guo W., Jiang J., Wang J. (2019). Enantioselective Synthesis of C-N Axially Chiral N-Aryloxindoles by Asymmetric Rhodium-Catalyzed Dual C-H Activation. Angew. Chem. Int. Ed..

[45] Zhang J., Simon M., Golz C., Alcarazo M. (2020). Gold-Catalyzed Atroposelective Synthesis of 1,1′-Binaphthalene-2,3′-Diols. Angew. Chem. Int. Ed..

[46] Shibata T., Sekine A., Mitake A., Kanyiva K.S. (2018). Intramolecular Consecutive Dehydro-Diels–Alder Eeaction for the Catalytic and Enantioselective Construction of Axial Chirality. Angew. Chem. Int. Ed..

[47] Cherney A.H., Kadunce N.T., Reisman S.E. (2015). Enantioselective and Enantiospecific Transition-Metal-Catalyzed Cross-Coupling Reactions of Organometallic Reagents to Construct C–C Bonds. Chem. Rev..

[48] Newton C.G., Wang S.G., Oliveira C.C., Cramer N. (2017). Catalytic Enantioselective Transformations Involving C–H Bond Cleavage by Transition-Metal Complexes. Chem. Rev..

[49] Liao G., Zhou T., Yao Q.J., Shi B.F. (2019). Recent Advances in the Synthesis of Axially Chiral Biaryls via Transition Metal-Catalysed Asymmetric C–H Functionalization. Chem. Commun..

[50] Liu C.X., Zhang W.W., Yin S.Y., Gu Q., You S.L. (2021). Synthesis of Atropisomers by Transition-Metal-Catalyzed Asymmetric C−H Functionalization Reactions. J. Am. Chem. Soc..

[51] Wang G.J., Huang J., Zhang J.M., Fu Z.Q. (2022). Catalytically Atroposelective Ring-Opening of Configurationally Labile Compounds to Access Axially Chiral Biaryls. Org. Chem. Front..

[52] Min X.L., Zhang X.L., Shen R., Zhang Q., He Y. (2022). Recent Advances in the Catalytic Asymmetric Construction of Atropisomers by Central-to-Axial Chirality Transfer. Org. Chem. Front..

[53] Yang H., Chen J., Zhou L. (2020). Construction of Axially Chiral Compounds via Central-to-Axial Chirality Conversion. Chem. Asian J..

[54] Quinonero O., Jean M., Vanthuyne N., Roussel C., Bonne D., Constantieux T., Bressy C., Bugaut X., Rodriguez J. (2016). Combining Organocatalysis with Central-to-Axial Chirality Conversion: Atroposelective Hantzsch-Type Synthesis of 4-Arylpyridines. Angew. Chem. Int. Ed..

[55] Zheng S.C., Wang Q., Zhu J.P. (2019). Catalytic Kinetic Resolution by Enantioselective Aromatization: Conversion of Racemic Intermediates of the Barton–Zard Reaction into Enantioenriched 3-Arylpyrroles. Angew. Chem. Int. Ed..

[56] Raut V.S., Jean M., Vanthuyne N., Roussel C., Constantieux T., Bressy C., Bugaut X., Bonne D., Rodriguez J. (2017). Enantioselective Syntheses of Furan Atropisomers by an Oxidative Central-to-Axial Chirality Conversion Strategy. J. Am. Chem. Soc..

[57] Bisag G.D., Pecorari D., Mazzanti A., Bernardi L., Fochi M., Bencivenni G., Bertuzzi G., Corti V. (2019). Central-to-Axial Chirality Conversion Approach Designed on Organocatalytic Enantioselective Povarov Cycloadditions: First Access to Configurationally Stable Indole–Quinoline Atropisomers. Chem. Eur. J..

[58] Li T.T., Mou C.L., Qi P.Y., Peng X.L., Jiang S.C., Hao G.F., Xue W., Yang S., Hao L., Chi Y.R. (2021). N-Heterocyclic Carbene-Catalyzed Atroposelective Annulation for Access to Thiazine Derivatives with C-N Axial Chirality. Angew. Chem. Int. Ed..

[59] Min X.L., Zhang X.L., Yi W.B., He Y. (2022). Brønsted Acid-Enhanced Copper-Catalyzed Atroposelective Cycloisomerization to Axially Chiral Arylquinolizones via Dearomatization of Pyridine. Nat. Commun..

[60] Wang Y.B., Zheng S.C., Hu Y.M., Tan B. (2017). Brønsted Acid-Catalysed Enantioselective Construction of Axially Chiral Arylquinazolinones. Nat. Commun..

[61] Zhang L., Zhang J., Ma J., Cheng D.J., Tan B. (2017). Highly Atroposelective Synthesis of Arylpyrroles by Catalytic Asymmetric Paal-Knorr Reaction. J. Am. Chem. Soc..

[62] Lv J., Zhang L., Luo S., Cheng J.-P. (2013). Switchable Diastereoselectivity in Enantioselective [4 + 2] Cycloadditions with Simple Olefins by Asymmetric Binary Acid Catalysis. Angew. Chem. Int. Ed..

[63] Zheng S.C., Wang Q., Zhu J.P. (2019). Catalytic Atropenantioselective Heteroannulation between Isocyanoacetates and Alkynyl Ketones: Synthesis of Enantioenriched Axially Chiral 3-Arylpyrroles. Angew. Chem. Int. Ed..

[64] He X.L., Zhao H.R., Song X., Jiang B., Du W., Chen Y.C. (2019). Asymmetric Barton−Zard Reaction to Access 3-Pyrrole-Containing Axially Chiral Skeletons. ACS Catal..

[65] Zhang X., Li S.N., Yu W.J., Xie Y.F., Tung C.H., Xu Z.H. (2022). Asymmetric Azide−Alkyne Cycloaddition with Ir(I)/Squaramide Cooperative Catalysis: Atroposelective Synthesis of Axially Chiral Aryltriazoles. J. Am. Chem. Soc..

[66] Guo W.T., Zhu B.H., Chen Y., Yang J., Qian P.C., Deng C., Ye L.W., Li L. (2022). Enantioselective Rh-Catalyzed Azide-Internal-Alkyne Cycloaddition for the Construction of Axially Chiral 1,2,3-Triazoles. J. Am. Chem. Soc..

[67] Zeng L.W., Li J.M., Cui S.L. (2022). Rhodium-Catalyzed Atroposelective Click Cycloaddition of Azides and Alkynes. Angew. Chem. Int. Ed..

[68] Kashima K., Teraoka K., Uekusa H., Shibata Y., Tanaka K. (2016). Rhodium-Catalyzed Atroposelective [2 + 2 + 2] Cycloaddition of Ortho-Substituted Phenyl Diynes with Nitriles: Effect of Ortho Substituents on Regio- and Enantioselectivity. Org. Lett..

[69] Sun L.C., Chen H.H., Liu B.X., Chang J.B., Kong L.H., Wang F., Lan Y., Li X.W. (2021). Rhodium-Catalyzed Atroposelective Construction of Indoles via C-H Bond Activation. Angew. Chem. Int. Ed..

[70] Ototake N., Morimoto Y., Mokuya A., Fukaya H., Shida Y., Kitagawa O. (2010). Catalytic Enantioselective Synthesis of Atropisomeric Indoles with an N−C Chiral Axis. Chem. Eur. J..

[71] Wang Y.C., Zhou X., Shan W.Y., Liao R.S., Deng Y.H., Peng F.Z., Shao Z.H. (2022). Construction of Axially Chiral Indoles by Cycloaddition−Isomerization via Atroposelective Phosphoric Acid and Silver Sequential Catalysis. ACS Catal..

[72] Wang P.Y., Huang Y.L., Jing J.R., Wang F., Li X.W. (2022). Rhodium (III)-Catalyzed Atroposelective Synthesis of C−N Axially Chiral Naphthylamines and Variants via C−H Activation. Org. Lett..

[73] He Y.P., Wu H., Wang Q., Zhu J.P. (2020). Palladium-catalyzed Enantioselective Cacchi Reaction: Asymmetric Synthesis of Axially Chiral 2,3-Disubstituted Indoles. Angew. Chem. Int. Ed..

[74] Tian M.M., Bai D.C., Zheng G.F., Chang J.B., Li X.W. (2019). Rh (III)-Catalyzed Asymmetric Synthesis of Axially Chiral Biindolyls by Merging C−H Activation and Nucleophilic Cyclization. J. Am. Chem. Soc..

[75] Li X.J., Zhao L.J., Qi Z.S., Li X.W. (2021). Construction of Atropisomeric 3-Arylindoles via Enantioselective Cacchi Reaction. Org. Lett..

[76] Wang C.S., Wei L., Fu C., Wang X.H., Wang C.J. (2021). Asymmetric Synthesis of Axially Chiral Naphthyl-C3-indoles via a Palladium-Catalyzed Cacchi Reaction. Org. Lett..

[77] Yang W.C., Chen B.X., Song K.L., Wu B., Gan W.E., Zheng Z.J., Cao J., Xu L.W. (2021). Pd-Catalyzed Enantioselective Tandem C−C Bond Activation/Cacchi Reaction between Cyclobutanones and o-Ethynylanilines. Org. Lett..

[78] Peng C., Kusakabe T., Kikkawa S., Mochida T., Azumaya I., Dhage Y.D., Takahashi K., Sasai H., Kato K. (2019). Asymmetric Cyclizative Dimerization of (ortho-Alkynyl Phenyl) (Methoxymethyl) Sulfides with Palladium (II) Bisoxazoline Catalyst. Chem. Eur. J..

[79] Zhang P., Xu Q., Wang X.M., Feng J., Lu C.J., Li Y.Z., Liu R.R. (2022). Enantioselective Synthesis of N−N Bisindole Atropisomers. Angew. Chem. Int. Ed..

[80] Chen K.W., Chen Z.H., Yang S., Wu S.F., Zhang Y.C., Shi F. (2022). Organocatalytic Atroposelective Synthesis of N–N Axially Chiral Indoles and Pyrroles by De Novo Ring Formation. Angew. Chem. Int. Ed..

[81] Gao Y.R., Wang L.Y., Zhang T., Yang B.M., Zhao Y. (2022). Atroposelective Synthesis of 1,1′-Bipyrroles Bearing a Chiral N-N Axis: Chiral Phosphoric Acid Catalysis with Lewis Acid Induced Enantiodivergence. Angew. Chem. Int. Ed..

[82] Han T.J., Zhang Z.X., Wang M.C., Xu L.P., Mei G.J. (2022). The Rational Design and Atroposelective Synthesis of Axially Chiral C2-Arylpyrrole-Derived Amino Alcohols. Angew. Chem. Int. Ed..

[83] Shao Y.D., Dong M.M., Wang Y.A., Cheng P.M., Wang T., Cheng D.J. (2019). Organocatalytic Atroposelective Friedländer Quinoline Heteroannulation. Org. Lett..

[84] Wan J.L., Liu H.X., Lan Y.J., Li X.H., Hu X.G., Li J., Xiao H.P., Jiang J. (2019). Catalytic Asymmetric Synthesis of Atropisomeric Quinolines through the Friedländer Reaction. Synlett.

[85] Shao Y.D., Han D.D., Ma W.Y., Cheng D.J. (2020). Chiral Phosphoric Acid Catalyzed Atroposelective and Diastereoselective Synthesis of 9-Aryltetrahydroacridines. Org. Chem. Front..

[86] Shao Y.D., Han D.D., Dong M.M., Yang X.R., Cheng D.J. (2021). A One-Pot Stepwise Approach to Axially Chiral Quinoline-3-Carbaldehydes Enabled by Iminium-Allenamine Cascade Catalysis. Org. Chem. Front..

[87] Zhang J., Xu Y., Wang Z.M., Zhong R., Wang Y.R. (2021). Organocatalyzed Cascade Aza-Michael/Aldol Reaction for Atroposelective Construction of 4-Naphthylquinoline-3-carbaldehydes. J. Org. Chem..

[88] Yang G.M., Sun S.F., Li Z.P., Liu Y.H., Wang J. (2021). Organocatalytic Atroposelective Heterocycloaddition to Access Axially Chiral 2-Arylquinolines. Commun. Chem..

[89] Cortright S.B., Johnston J.N. (2002). IAN-Amines: Direct Entry to a Chiral C2-Symmetric Zirconium (IV) β-Diketimine Complex. Angew. Chem. Int. Ed..

[90] Zhang L., Shen J.H., Wu S., Zhong G.F., Wang Y.B., Tan B. (2020). Design and Atroposelective Construction of IAN analogues by Organocatalytic Asymmetric Heteroannulation of Alkynes. Angew. Chem. Int. Ed..

[91] Yang G.M., Li Z.P., Liu Y.H., Guo D.H., Sheng X.J., Wang J. (2021). Organocatalytic Higher-Order [8 + 2] Cycloaddition for the Assembly of Atropoenantiomeric 3-Arylindolizines. Org. Lett..

[92] Ma R., Wang X.X., Zhang Q.Y., Chen L., Gao J., Feng J., Wei D.H., Du D. (2021). Atroposelective Synthesis of Axially Chiral 4-Aryl α-Carbolines via N-Heterocyclic Carbene Catalysis. Org. Lett..

[93] Yang H., Sun H.R., He R.Q., Yu L., Hu W., Chen J., Yang S., Zhang G.G., Zhou L. (2022). Organocatalytic Cycloaddition of Alkynylindoles with Azonaphthalenes for Atroposelective Construction of Indole-based Biaryls. Nat Commun..

[94] Xu W.L., Zhao W.M., Zhang R.X., Chen J., Zhou L. (2021). Organocatalytic Cycloaddition–Elimination Cascade for Atroposelective Construction of Heterobiaryls. Chem. Sci..

[95] Zhang C.L., Gao Y.Y., Wang H.Y., Zhou B.A., Ye S. (2021). Enantioselective Synthesis of Axially Chiral Benzothiophene/Benzofuran-Fused Biaryls by N-Heterocyclic Carbene Catalyzed Arene Formation. Angew. Chem. Int. Ed..

[96] Wang L., Zhong J.L., Lin X.F. (2019). Asymmetric Phosphoric Acid-Catalyzed Atroposelective Three Component Cascade Reaction: Highly Enantioselective Synthesis of Axially Chiral N-Arylindoles. Angew. Chem. Int. Ed..

[97] Man N.N., Lou Z.B., Li Y.M., Yang H.J., Zhao Y.F., Fu H. (2020). Organocatalytic Atroposelective Construction of Axially Chiral N-Aryl Benzimidazoles Involving Carbon−Carbon Bond Cleavage. Org. Lett..

[98] Chang Y., Xie C.D., Liu H., Huang S.L., Wang P.F., Qin W.L., Yan H.L. (2022). Organocatalytic Atroposelective Construction of Axially Chiral N, N- and N, S-1,2-Azoles Through Novel Ring Formation Approach. Nat Commun..

[99] Arae S., Beppu S., Kawatsu T., Igawa K., Tomooka K., Irie R. (2018). Asymmetric Synthesis of Axially Chiral Benzocarbazole Derivatives Based on Catalytic Enantioselective Hydroarylation of Alkynes. Org. Lett..

[100] Liu Y.D., Wu X.Y., Li S., Xue L., Shan C.H., Zhao Z.X., Yan H.L. (2018). Organocatalytic Atroposelective Intramolecular [4 + 2] Cycloaddition: Synthesis of Axially Chiral Heterobiaryls. Angew. Chem. Int. Ed..

[101] Peng L., Li K., Xie C.D., Li S., Xu D., Qin W.L., Yan H.L. (2019). Organocatalytic Asymmetric Annulation of ortho-Alkynylanilines: Synthesis of Axially Chiral Naphthyl-C2-indoles. Angew. Chem. Int. Ed..

[102] Xu D., Huang S.L., Hu F.L., Peng L., Jia S.Q., Mao H., Gong X.N., Li F.L., Qin W.L., Yan H.L. (2022). Diversity-Oriented Enantioselective Construction of Atropisomeric Heterobiaryls and N-Aryl Indoles. CCS Chem..

[103] Wang Z.S., Zhu L.J., Li C.T., Liu B.Y., Hong X., Ye L.W. (2022). Synthesis of Axially Chiral N-Arylindoles via Atroposelective Cyclization of Ynamides Catalyzed by Chiral Brønsted Acids. Angew. Chem. Int. Ed..

[104] Kwon Y., Li J.Q., Reid J.P., Crawford J.M., Jacob R., Sigman M.S., Toste D.F., Miller S.J. (2019). Disparate Catalytic Scaffolds for Atroposelective Cyclodehydration. J. Am. Chem. Soc..

